# Disentangling the individual and contextual effects of math anxiety: A global perspective

**DOI:** 10.1073/pnas.2115855119

**Published:** 2022-02-07

**Authors:** Nathan T. T. Lau, Zachary Hawes, Paul Tremblay, Daniel Ansari

**Affiliations:** ^a^Department of Psychology and Faculty of Education, University of Western Ontario, London, ON N6A 3K7, Canada;; ^b^Applied Psychology and Human Development, Ontario Institute for Studies in Education, University of Toronto, Toronto, ON M5S 1V6, Canada;; ^c^Department of Psychology, University of Western Ontario, London, ON N6A 3K7, Canada

**Keywords:** math anxiety, math achievement, contextual effect, international assessments

## Abstract

Math anxiety is a common affective disorder in students that is characterized by intrusive thoughts that disrupt critical cognitive resources required for math problem-solving. Consistent associations between math anxiety and math achievement have been observed across countries and age groups, placing math anxiety among other important correlates of math achievement, such as socioeconomic status and magnitude representation ability. However, studies examining math anxiety’s relation to achievement have largely focused on the effect of students’ own math anxiety (individual effect), while little is known regarding the effect of math anxiety in students’ educational context (contextual effect). Using three international studies of achievement (*n* = 1,175,515), we estimated both the individual and contextual effects of math anxiety across the globe. Results suggest that while there are consistent individual effects in virtually all countries examined, the contextual effects are varied, with only approximately half of the countries exhibiting a contextual effect. Additionally, we reveal that teacher confidence in teaching math is associated with a reduction of the individual effect, and country’s level of uncertainty avoidance is related to a lessening of the contextual effect. Finally, we uncovered multiple predictors of math anxiety; notably, student perception of teacher competence was negative related with math anxiety, and parental homework involvement was positively related with math anxiety. Taken together, these results suggest that there are significant between-country differences in how math anxiety may be related with math achievement and suggest that education and cultural contexts as important considerations in understanding math anxiety’s effects on achievement.

## Significance

Using three large-scale international assessments of student achievement, the current study examined the antecedents of math anxiety and the relation between math anxiety and math achievement across the globe. Results suggest that individual math anxiety is negatively associated with math achievement across the globe. Importantly, we uncovered a contextual effect of math anxiety where the level of math anxiety in one’s educational peer group predicts math achievement above and beyond what could be predicted by one’s own math anxiety. Further, there is significant between-country variability in this contextual effect—only half of the examined countries’ contextual effect was statistically significant. Our results reveal an effect of educational peer’s math anxiety on math achievement and reinforce extant research findings.

Math anxiety is the “feeling of tension, apprehension or even dread, that interferes with the ordinary manipulation of numbers and the solving of mathematical problems” (1). Consistent and robust associations have been demonstrated between math anxiety and math achievement, indicating that people with higher feelings of fear and anxiety toward math tend to have lower math achievement (2–5). Results from a recent meta-analysis (3) estimate the effect size to be *r* = 0.28, a small to moderate effect size, comparable with the effects of other important correlates of math achievement, including magnitude processing skills (*r* = 0.24) (6) and socioeconomic status (*r* = 0.35) (7).

High prevalence rates of math anxiety have been reported across countries (8) and age groups (5, 9), and the consequences of math anxiety are far reaching. People with heightened levels of math anxiety often experience a lifelong tendency to avoid math, math-related situations, career paths that require math, and most notably, courses and degrees in Science, Technology, Engineering, and Mathematics (10–12). In short, there is evidence that math anxiety negatively impacts math performance and can influence how one experiences and interacts with the world.

An in-depth examination of the association between math anxiety and math achievement suggests that math anxiety is detrimental to performance in many school-related math skills, including problem-solving (4), simple arithmetic (1), and basic number processing (13). The most influential account of the mechanism behind these associations posits that math anxiety interferes with math performance by compromising cognitive resources that are key for success in math. Specifically, worries and intrusive thoughts evoked by math anxiety disrupt and compete for cognitive resources, such as working memory, that are vital for math problem-solving (14). Both behavioral (1, 9, 15–17) evidence and neuroimaging (18–20) evidence have been forwarded in support of this interference account.

While the mechanism that underlies the link between math anxiety and math achievement has largely been conceptualized as a within-individual phenomenon, studies that examine the causes of math anxiety have found a multitude of diverse correlates of math anxiety whose origins span well beyond the individual. These correlates can largely be categorized into individual factors, interpersonal factors, and environmental factors.

Individual factors include genetics (21), working memory capacity (17), attentional bias (22), and affective or physiological responses (23). Interpersonal factors include parental support and expectations (9), parental attitudes toward mathematics (24), teacher’s own math anxiety (25, 26), teacher self-efficacy (27), and teacher expectations of students (28). Environmental factors include math activities at home (29), classroom atmosphere (30), and cultural background (31).

The abovementioned studies have markedly increased understanding of the individual and environmental variables that contribute to math anxiety and of the mechanisms through which math anxiety affects math achievement. However, these studies are nevertheless limited in three important and interconnected aspects that have stifled understanding of math anxiety.

First, the extant literature examining math anxiety has largely operated in separate information silos with little cross-talk; this consequently has left several important questions unanswered. Specifically, while multiple individual, interpersonal, and environmental factors have been found to be related to math anxiety, most studies that examine these individual and environmental factors have done so in relative isolation of other proposed predictors of math anxiety. For example, the basic numerical magnitude processing predictors of math anxiety have not been studied in tandem with interpersonal factors, such as teachers’ math anxiety. As such, little is known regarding whether any of the known individual, interpersonal, and environmental predictors would uniquely predict math anxiety after controlling for other proposed predictors of math anxiety. Moreover, studies that examine, on the one hand, the predictors of math anxiety and on the other, the relationship between math anxiety and math achievement have also been conducted in isolation of one another. As such, it is unknown whether the identified individual, interpersonal, and environmental predictors of math anxiety may serve as unobserved confounders that may inflate the relation between math anxiety and math achievement.

Second, perhaps as a consequence of information silos in the extant literature, the multilevel nature of math anxiety has been scarcely explored. Particularly, hints of the multilevel nature of math anxiety can be found from studies that showed that multiple interpersonal and environmental predictors of math anxiety (e.g., teacher’s own math anxiety) (25, 26) exist at a higher, clustered level (i.e., these factors affect a group of individuals who shared the same education environment rather than specific individuals). This suggests there may be systematic variations of math anxiety at the education environment level, and student membership in any particular education environment would be predictive of student math anxiety to some degree (*Methods* has reasons why we elected to use the broader term education environment to refer to influences from the school or classroom).

Importantly, when considering math anxiety as a predictor, the variability of math anxiety at the individual level and the variability of math anxiety at the education environment level may have independent and different effects on student math achievement. To see why this might be the case, it is useful to envision a scenario where a child with some degree of math anxiety is put into an education environment with low average math anxiety and an alternative scenario where the same child is put into an education environment with high average math anxiety. Irrespective of the cause of a particular environment’s average level of math anxiety (e.g., teacher’s own math anxiety) (25, 26), it is possible that the environment-average level of math anxiety could serve to predict the child’s math achievement over and above what could be predicted by the child’s own level of math anxiety.

Put more formally, the effect that the education environment–average math anxiety has on individual math achievement is a contextual effect. A contextual effect is said to occur if an aggregation (i.e., mean) of an individual-level variable at a higher level (i.e., aggregating individual math anxiety by calculating classroom average math anxiety) makes an independent contribution to explaining the outcome variable over and above the contribution of the same variable at the lower level (i.e., individual math anxiety). In this case where math anxiety is used to predict math achievement, a contextual effect of math anxiety at the education environment level is the additional variance of math achievement explained by education environment–average math anxiety over and above the variance explained by individual math anxiety.

Decomposing the total effect of math anxiety on math achievement into the individual effect and contextual effect allows for a more accurate account of how math anxiety affects math performance. Indeed, the exploration of the potential of contextual effects is especially important, as it is possible for contextual effects to significantly differ in both size and direction from the individual effect. For instance, research on the big fish, little pond effect (a review is in ref. 32) has revealed that while the relation between achievement and self-efficacy may be positive at the individual level (i.e., the better my grades, the more confident I am in my abilities), the relation is reversed at the classroom level (i.e., the better my peer’s average grades, the less confident I am in my abilities).

To date, few studies (e.g., refs. 25 and 26) have accounted for the multilevel nature of math anxiety, and to our knowledge, no studies thus far have examined the possibility of a contextual effect of math anxiety at the education environment level. Failing to account for the multilevel nature of math anxiety is problematic statistically, theoretically, and practically. Statistically, members in the same group are more likely to be similar than members from a different group. Analyses that ignore this grouping structure violate the assumption of independence, thereby increasing the risk of yielding misleading parameter estimates, SE estimates, and fit indices and the risk of type I error (33, 34). Theoretically, as it is possible for individual and contextual effects to differ in both strength and direction, conclusions drawn using analyses that ignore the nested nature of the data are incomplete and often misleading (35). Practically, as no study thus far has examined the size and direction of the contextual effect at the education environment, the potential utility of interventions for the alleviation or prevention of the adverse effects of math anxiety at different levels (e.g., individual intervention vs. classroom intervention) is currently unknown.

Third and finally, the overrepresentation of participants from Western, educated, industrialized, rich, and democratic (WEIRD) societies is a prevalent and important problem afflicting research in psychology (36). For instance, in one study, it was found that over 96% of subjects in papers published in top psychological journals belong to WEIRD societies (37). This problem similarly applies to the extant literature on math anxiety; for instance, ∼80% of the 747 effect sizes examined in the meta-analysis by Barroso et al. (3) on the relation between math anxiety and math achievement were from the European and North American continents. Consequently, much is still unknown regarding math anxiety in other societies.

Nonetheless, even with the small proportion of effect sizes from non-Western countries, Barroso et al. (3) have found some provisional evidence to suggest that the relation between math anxiety and math achievement may differ between continents and regions. Similarly, Foley et al. (31) found that different countries have varying levels of math anxiety and that this variability in country-average math anxiety is negatively related with math achievement. It has been proposed that between-country differences in societal pressure placed on the student, cultural norms associated with schooling, and expectations of the type and amount of support that children are provided at home may be possible reasons for the observed cross-national variability (31).

When these findings are viewed from a multilevel modeling perspective, however, it is apparent that these studies are somewhat underspecified as they do not break down the observed total effect of math anxiety on math achievement into its individual effect and contextual effect constituents. Consequently, multiple important and unanswered question arises. Specifically, it is currently an open question whether there may be between-country differences in the magnitude of the individual effect (i.e., the amount of increase or decrease in math achievement per unit increase in individual math anxiety) and the contextual effect (i.e., the amount of increase or decrease in math achievement per unit increase in education environment–average math anxiety). Relatedly, it remains poorly understood whether cultural differences between countries may explain these variations.

Additionally, similar to the contextual effect of math anxiety at the education environment level, it is also possible that there is a contextual effect of math anxiety at the country level. Going back to the hypothetical situation outlined above, a contextual effect of math anxiety at the country level would entail a scenario where the same child is put into a low average math anxiety country and an alternative scenario where the same child is put into a high average math anxiety country. If the country’s average level of math anxiety is predictive of math achievement beyond what could be accounted for by the child’s individual math anxiety, there would be a contextual effect of math anxiety at the country level.

Thus far, only a few studies in the literature have systematically explored the possibility of between-country differences in math anxiety (8, 31). Critically, no studies have explored between-country differences in the individual and contextual effects of math anxiety.

To fill in the research gap identified above, the current study utilized a multilevel structural equation modeling approach in order to model the relations between math anxiety, math achievement, and their predictors. The current study draws on data from three large-scale international studies of student achievement: Trends in International Mathematics and Science Study 2015 (TIMSS) Grade 4 and Grade 8 (38) and the Program for International Student Assessment 2012 Grade 8 (PISA) (39). This sample is larger and more diverse than any other math anxiety study to date. The current study extends existing research in three important ways.

1) Given the dearth of research that examines the relation between math anxiety and math achievement under a multilevel modeling framework, we investigate whether the aggregation of math anxiety at the education environment (i.e., education environment–average math anxiety) makes an independent contribution to explaining math achievement over and above the contribution of individual math anxiety. Further, few extant studies have systematically examined the potential of between-country differences in the individual and contextual effects. The exploration of possible between-country differences in the individual and contextual effects in the current study is important, as it would highlight the degree to which research findings can be applied in different cultural contexts.
2) Extant research on the predictors of math anxiety has been done in relative isolation. For instance, the individual predictors of math anxiety (e.g., ref. 40) have typically been studied separately from environmental predictors of math anxiety, such as the home environment (e.g., ref. 24). As the TIMSS Grade 4 database includes a rich set of potentially relevant predictors of math anxiety (*Methods*), we investigated the relative strength of influence of these variables. In doing so, we consolidate current research findings and provide a more coherent picture of how different variables may predict math anxiety.
3) Since many factors have been found to predict math anxiety, it is important to examine how these predictors may affect the relationship between math anxiety and math achievement. Including these predictors alongside the estimation of math anxiety’s effect on math achievement would control for potential shared variance explained between the predictors and math anxiety. We, therefore, explore whether any of these variables may serve as an unobserved confounder that inflates the relation between math anxiety and math achievement.

## Results

Results most pertinent to addressing the three research questions are presented below. *SI Appendix* has descriptions and results of additional analyses. Please note that L1, L2, and L3 refer to the individual level, the education environment level, and the country level, respectively. *Methods* has more details.

### Is There a Contextual Effect of Math Anxiety at the Education Environment Level?

To establish a baseline model, a total sample model was computed while ignoring country membership. In the total sample of TIMSS Grade 4, L1 individual math anxiety was negatively related to math achievement $\left(\pi_{1,j,k}=\;-0.220,SE=0.004,\;P<0.001,\;\text{Δ}=\;0.524\right)$, and the contextual effect of L2 math anxiety was also negative $\left(\beta_{0,0,k}=-0.161,\;SE=0.012,P<0.001,\text{Δ}=\;0.445\right)$. In the total sample of TIMSS Grade 8, L1 individual math anxiety was negatively related to math achievement $\left(\pi_{1,j,k}=\;-0.169,\;SE=0.003,\;P<0.001,\text{Δ}=\;0.437\right)$, and the contextual effect of L2 math anxiety was also negative $\left(\beta_{0,0,k}=-0.137,\;SE=0.011,\;P<0.001,\text{Δ}=\;0.411\right)$. Finally, in the total sample of PISA, L1 individual math anxiety was negatively related to math achievement $\left(\pi_{1,j,k}=\;-0.150,\;SE=0.003,\;P<0.001,\;\text{Δ}=\;0.390\right)$, and the contextual effect of L2 math anxiety was also negative $\left(\beta_{0,0,k}=-0.105,\;SE=0.018,\;P<0.001,\text{Δ}=\;0.380\right)$.

In sum, all three databases yielded negative L1 and L2 associations between math anxiety and math achievement with comparable effect sizes. The negative association between math anxiety and math achievement at L1 suggests that students with higher math anxiety tend to have lower math achievement, while the negative association at L2 suggests that when individual differences in math anxiety are controlled, education environment–average math anxiety has a negative effect on math achievement. The effect size of the individual and contextual effects ranged from 0.380 to 0.524, indicating a small to medium effect size (41).

### Are There Between-Country Differences in the Individual and Contextual Effects?

To examine the generalizability of the baseline model, a multigroup analysis was conducted with country membership as the grouping variable. To test whether there are statistically significant between-country differences in the magnitude of the L1 individual effect and L2 contextual effect, a constrained model—in which the L1 individual effect and L2 contextual effect are held constant across countries—was first estimated for each database. Model fit of the constrained models was poor for all three databases (TIMSS Grade 4: χ^2^ = 7878.10, degrees of freedom [df] = 318, Comparative Fit Index [CFI] = 0.603, Tucker–Lewis Index [TLI] = 0.863, rms error of approximation [RMSEA] = 0.056; TIMSS Grade 8: χ^2^ = 6987.58, df = 379, CFI = 0.182, TLI = 0.724, RMSEA = 0.048; PISA: χ^2^ = 10358.80, df = 270, CFI = 0.052, TLI = 0.677, RMSEA = 0.077), indicating that the restriction of the individual and contextual effects being equal across countries may have significantly reduced model fit.

**Fig. 1.**
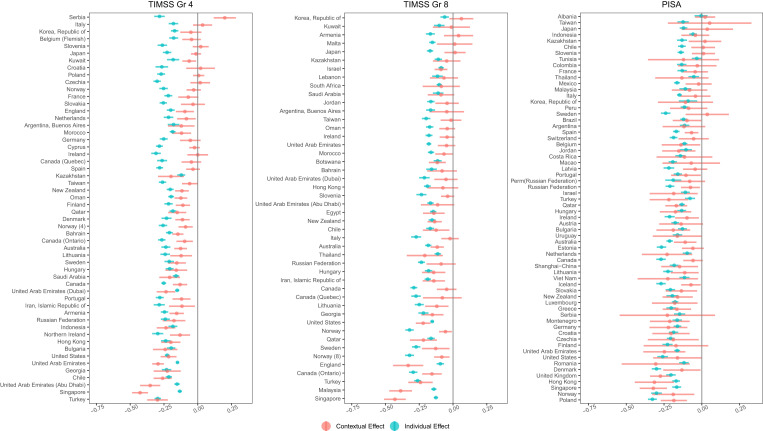
Individual and contextual effects of math anxiety on math achievement. Countries are ordered according to the average of the two parameters. CIs reflect an *α* of 0.05. As such, parameters are statistically significant if the 95% CI does not cross zero (drawn in black).

Given results suggesting that both individual and contextual effects differ between countries, an unconstrained model in which both L1 individual effect and L2 contextual effect are free to vary across countries was next estimated for each database. For the unconstrained model, country-specific L1 and L2 parameters are presented in Fig. 1 (*SI Appendix* has coefficients and effect sizes).

For the TIMSS Grade 4, the L1 individual effects of math anxiety on math achievement were statistically significant for all countries and ranged from −0.309 to −0.122. The L2 contextual effect between math anxiety and math achievement was statistically significant in 38 of the 54 countries and ranged from −0.427 to 0.200. All statistically significant L2 contextual effects were negative except for Serbia, which had a statistically significant positive effect. For the TIMSS Grade 8, L1 individual effect of math anxiety on math achievement was statistically significant for all countries and ranged from −0.322 to −0.062. The L2 contextual effect of math anxiety on math achievement was statistically significant in 22 of the 46 countries and ranged from −0.430 to 0.062. All statistically significant L2 contextual effects were negative. For the PISA, L1 individual effect of math anxiety on math achievement was statistically significant for all countries except Albania and ranged from −0.327 to −0.005. The L2 contextual effect of math anxiety on math achievement was statistically significant in 35 of the 64 countries and ranged from −0.321 to 0.052. All statistically significant L2 contextual effects were negative.

In sum, results from the constrained model suggest that there are statistically significant between-country differences in both the individual and contextual effects and that the relations between the variables from one country cannot be readily generalized to another. Results from the unconstrained model are consistent across databases. Specifically, we observed a robust negative effect between individual math anxiety and math achievement in all countries except one country. However, the L2 contextual effect is more tenuous, with only 47 to 70% of the countries exhibiting a statistically significant negative contextual effect.

### What Individual and Environmental Factors Predict Math Anxiety?

**Fig. 2.**
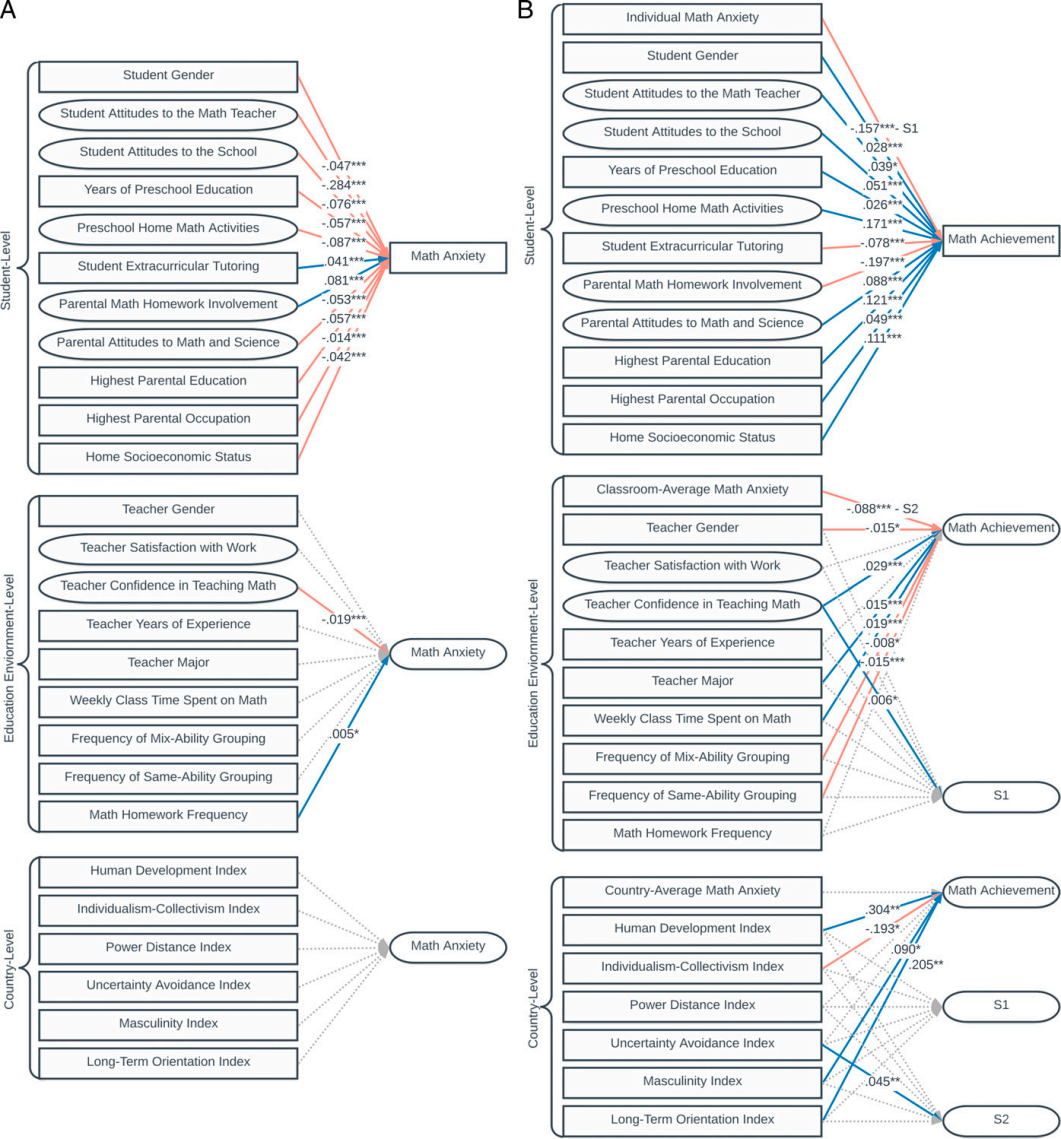
Results for the three-level models. (*A*) The three-level model with math anxiety as the outcome. (*B*) The three-level model with random slopes with math achievement as the outcome. Rectangles are observed variables, and rounded rectangles are latent variables. S1 refers to the random slope of individual math anxiety to math achievement, and S2 refers to the random slope of education environment math anxiety to math achievement. Red lines refer to negative relations; blue lines refer to positive relations.

To examine the individual and environmental factors that predict math anxiety, we estimated a three-level model with math anxiety as the outcome variable. Due to missing data in the TIMSS Grade 8 and PISA databases, this analysis was solely conducted using the TIMSS Grade 4 database (*Methods* has more details). Results are presented in Fig. 2*A* and Table 1. Results indicate that a multitude of variables at L1 is associated with math anxiety. The strongest predictor of math anxiety at L1 was students’ attitudes toward the math teacher $\left[\pi_{2,j,k}=\;-0.284\left(0.029\right),P<0.001,\;\Delta =\;0.274\right]$. Specifically, students’ attitudes toward the math teacher’s competence and fairness are positively related with lowered math anxiety. At L2, only two variables were significantly related with math anxiety. Specifically, teachers’ confidence in teaching math is associated with a reduction in student math anxiety $\left[\beta_{0,3,k}=\;-0.019\left(0.004\right),P<0.001,\Delta =\;0.028\right]$, and homework frequency is positively associated with increased math anxiety $\left[\pi_{2,j,k}=\;-0.005\left(0.003\right),P=\;0.048,\Delta =\;0.010\right]$.

**Table 1.** Results for the three-level model with math anxiety as the outcome variable

Fixed effects	Coefficient (SE)	Effect size (Δ)
Level 1		
Student gender, $\pi_{1,j,k}$	–0.047 (0.009)***	0.099
Student attitudes toward the math teacher, $\pi_{2,j,k}$	–0.284 (0.029)***	0.274
Student attitudes toward the school, $\pi_{3,j,k}$	–0.076 (0.014)***	0.100
Student years of preschool education, $\pi_{4,j,k}$	–0.013 (0.004)***	0.027
Student preschool home mathematics activities, $\pi_{5,j,k}$	–0.087 (0.009)***	0.099
Student current extracurricular tutoring/lessons, $\pi_{6,j,k}$	0.041 (0.007)***	0.086
Parental involvement in mathematics homework, $\pi_{7,j,k}$	0.081 (0.009)***	0.126
Parental attitudes toward mathematics and science, $\pi_{8,j,k}$	–0.053 (0.007)***	0.068
Parents’ highest education level, $\pi_{9,j,k}$	–0.057 (0.004)***	0.119
Parents’ occupation, $\pi_{10,j,k}$	–0.014 (0.003)***	0.029
Home socioeconomic status, $\pi_{11,j,k}$	–0.042 (0.005)***	0.088
Level 2		
Teacher gender, $\beta_{0,1,k}$	0.011 (0.008)	0.023
Teacher satisfaction with work, $\beta_{0,2,k}$	–0.005 (0.005)	0.008
Teacher confidence in teaching mathematics, $\beta_{0,3,k}$	–0.019 (0.004)***	0.028
Teacher years of experience, $\beta_{0,4,k}$	–0.006 (0.004)	0.013
Teacher major, $\beta_{0,5,k}$	–0.001 (0.005)	0.002
Weekly class time spent on mathematics, ${\;\beta }_{0,6,k}$	–0.001 (0.003)	0.002
Frequency of mixed-ability grouping, $\beta_{0,7,k}$	0.003 (0.003)	0.006
Frequency of same-ability grouping, $\beta_{0,8,k}$	0.000 (0.003)	0.000
Mathematics homework frequency, $\beta_{0,9,k}$	0.005 (0.003)*	0.010
Level 3		
Average initial math anxiety, $\gamma_{0,0,0}$	–0.013 (0.028)	—
United Nations Human Development Index, $\gamma_{0,0,1}$	0.041 (0.040)	0.086
Individualism—collectivism, $\gamma_{0,0,2}$	–0.084 (0.057)	0.176
Power distance, $\gamma_{0,0,3}$	0.005 (0.005)	0.010
Uncertainty avoidance, $\gamma_{0,0,4}$	0.007 (0.038)	0.015
Masculinity, $\gamma_{0,0,5}$	0.052 (0.048)	0.109
Long-term orientation, $\gamma_{0,0,6}$	–0.065 (0.040)	0.136
Random effects		
Math anxiety L1 residual, $e_{i,j,k}$	0.873***	—
Math anxiety L2 residual, $\;r_{0,j,k}$	0.034***	—
Math anxiety L3 residual, ${\;\mu }_{0,0,k}$	0.041***	—

*P < 0.05; ***P < 0.001.

### Do the Predictors of Math Anxiety Serve as Unobserved Confounders to the Relation between Math Anxiety and Math Achievement?

In the final analysis, the relation between math anxiety and math achievement while controlling for the individual and environmental predictors of math anxiety was explored. To this end, both math anxiety and individual and environmental factors were included as predictors of math achievement. Further, the random slopes were also regressed onto these predictors as a test of cross-level interactions. Results are presented in Fig. 2*B* and Table 2. Results indicate that after accounting for a large repertoire of potential covariates, the average L1 individual effect $\left(\gamma_{1,0,0}\right)$ and the average L2 contextual effect $\left(\gamma_{0,1,0}\right)$ remain robust. The average effect size for the individual effect was 0.491, and the average effect size for the L2 contextual effect was 0.312, a small to medium effect size.

**Table 2.** Results for the three-level model with random slopes with covariates for TIMSS Grade 4

Fixed effects	Coefficient (SE)	Effect size (Δ)
Model math achievement, $\pi_{0jk}$		
Level 1		
Student gender, $\pi_{2jk}$	0.028 (0.004)***	0.085
Student attitudes toward the math teacher, $\pi_{3,j,k}$	0.039 (0.017)*	0.054
Student attitudes toward the school, $\pi_{4,j,k}$	0.051 (0.012)***	0.096
Student years of preschool education,$\;\pi_{5,j,k}$	0.026 (0.004)***	0.079
Student preschool home math activities, $\pi_{6,j,k}$	0.171 (0.010)***	0.281
Student current extracurricular tutoring/lessons, $\pi_{7,j,k}$	–0.078 (0.013)***	0.236
Parental involvement in math homework, $\pi_{8,j,k}$	–0.197 (0.010)***	0.472
Parental attitudes toward math and science, $\pi_{9,j,k}$	0.088 (0.007)***	0.164
Parents’ highest education level, $\pi_{10,j,k}$	0.121 (0.007)***	0.366
Parents’ occupation, $\pi_{11,j,k}$	0.049 (0.004)***	0.148
Home socioeconomic status, $\pi_{12,j,k}$	0.111 (0.009)***	0.336
* *Level 2		
Teacher gender, $\beta_{0,2,k}$	–0.015 (0.007)*	0.045
Teacher satisfaction with work, $\beta_{0,3,k}$	0.010 (0.006)	0.023
Teacher confidence in teaching math, $\beta_{0,4,k}$	0.029 (0.006)***	0.061
Teacher years of experience, $\beta_{0,5,k}$	0.010 (0.008)	0.030
Teacher major, $\beta_{0,6,k}$	0.015 (0.004)***	0.048
Weekly class time spent on math, $\beta_{0,7,k}$	0.019 (0.005)***	0.058
Frequency of mixed-ability grouping, $\beta_{0,8,k}$	–0.008 (0.004)*	0.024
Frequency of same-ability grouping, $\beta_{0,9,k}$	–0.015 (0.004)***	0.045
Math homework frequency, $\beta_{0,10,k}$	0.004 (0.004)	0.012
* *Level 3		
Average initial math achievement, $\gamma_{0,0,0}$	–0.008 (0.055)	—
Country math anxiety, $\gamma_{0,0,1}$	0.032 (0.044)	0.097
United Nations Human Development Index, $\gamma_{0,0,2}$	0.304 (0.105)**	0.921
Individualism–collectivism, $\gamma_{0,0,3}$	–0.193 (0.082)*	0.585
Power distance, $\gamma_{0,0,4}$	–0.053 (0.137)	0.161
Uncertainty avoidance, $\gamma_{0,0,5}$	–0.066 (0.077)	0.200
Masculinity, $\gamma_{0,0,6}$	0.090 (0.045)*	0.273
Long-term orientation, $\gamma_{0,0,7}$	0.205 (0.078)**	0.621
Model random slope of individual math anxiety on math achievement, $\pi_{1jk}$		
* *Level 2		
Teacher gender, $\beta_{1,1,k}$	–0.001 (0.002)	—
Teacher satisfaction with work, $\beta_{1,2,k}$	–0.001 (0.003)	—
Teacher confidence in teaching math, $\beta_{1,3,k}$	0.006 (0.003)*	—
Teacher years of experience, $\beta_{1,4,k}$	0.001 (0.002)	—
Teacher major, $\beta_{1,5,k}$	0.001 (0.002)	—
Weekly class time spent on math, $\beta_{1,6,k}$	0.002 (0.002)	—
Frequency of mixed-ability grouping, $\beta_{1,7,k}$	–0.002 (0.002)	—
Frequency of same-ability grouping, $\beta_{1,8,k}$	0.002 (0.002)	—
Math homework frequency, $\beta_{1,9,k}$	0.001 (0.002)	—
* *Level 3		
Individual math anxiety intercept, $\gamma_{1,0,0}$	–0.157 (0.005)***	0.476
United Nations Human Development Index, $\gamma_{1,0,1}$	0.013 (0.009)	—
Individualism–collectivism, $\gamma_{1,0,2}$	–0.006 (0.009)	—
Power distance, $\gamma_{1,0,3}$	0.005 (0.009)	—
Uncertainty avoidance, $\gamma_{1,0,4}$	0.003 (0.007)	—
Masculinity, $\gamma_{1,0,5}$	0.000 (0.006)	—
Long-term orientation, $\gamma_{1,0,6}$	0.010 (0.005)	—
Model random slope of education environment–average math anxiety on math achievement, $\pi_{1jk}$		
* *Level 3		
Education environment math anxiety intercept, $\gamma_{0,1,0}$	–0.088 (0.014)***	0.267
United Nations Human Development Index, $\gamma_{0,1,1}$	0.017 (0.015)	—
Individualism–collectivism, $\gamma_{0,1,2}$	0.016 (0.019)	—
Power distance, $\gamma_{0,1,3}$	–0.009 (0.021)	—
Uncertainty avoidance, $\gamma_{0,1,4}$	0.045 (0.015)**	—
Masculinity, $\gamma_{0,1,5}$	0.001 (0.011)	—
Long-term orientation, $\gamma_{0,1,6}$	0.004 (0.010)	—
Random effects		
Math achievement L1 residual, $e_{i,j,k}$	0.374 (0.012)***	—
Math achievement L2 residual, $\;r_{0,j,k}$	0.130 (0.016)***	—
Math achievement L3 residual, ${\;\mu }_{0,0,k}$	0.109 (0.027)***	—
Individual math anxiety at L2 residual, ${\text{r}}_{1,j,k}$	0.001 (0.000)**	—
Individual math anxiety at L3 residual, ${\text{u}}_{1,0,k}$	0.001 (0.000)***	—
Education environment math anxiety at L3 residual, $u_{0,1,k}$	0.005 (0.001)***	—

*P < 0.05; **P < 0.01; ***P < 0.001.

Further, a cross-level interaction was found in which the greater the teacher’s confidence in teaching math, the lesser the magnitude of individual math anxiety’s effect on math achievement $\left(\beta_{1,3,k}\right)$. This suggests that teachers with higher confidence in teaching math may be associated with a milder effect of individual math anxiety on math achievement. Similarly, it was found that a country’s degree of uncertainty avoidance is related to the degree to which education environment–average math anxiety is related with math achievement $\left(\gamma_{0,1,4}\right)$. Specifically, countries with higher levels of uncertainty avoidance tend to have education environments in which average math anxiety does not affect student math achievement as severely. Finally, while there are significant variations in the magnitude of the individual effect of math anxiety on math achievement at L3 $\left({\text{u}}_{1,0,k}\right)$, none of the country-level predictors significantly predict the variability.

## Discussion

In recent decades, math anxiety has received increasing research attention both as a predictor of math achievement and as a phenomenon to be studied and mitigated. Despite this increased attention, the extant literature has remained largely an endeavor of understanding the effects of math anxiety as an individual-level phenomenon. However, it is becoming increasingly clear that math anxiety occurs within a complex ecosystem that includes predictors within multiple nested levels. Using multiple large-scale international studies of student achievement, the current study presents the most comprehensive examination of 1) the relation between math anxiety and math achievement across the globe, 2) the individual and environmental predictors of math anxiety, and 3) any potential factors that could explain the relation between math anxiety and math achievement. Multiple important findings were uncovered in the current study.

### Summary of Pertinent Findings.

Research over the past few decades has revealed multiple predictors of math anxiety that can be found at the level of the individual [e.g., gender (42), student attitude toward the math teacher (26), etc.] as well as at the level of the education environment [e.g., teacher math anxiety (43), classroom atmosphere (44), etc.]. However, until now, the different levels at which predictors of math anxiety can be identified have largely been studied in isolation of one another. To the best of our knowledge, the current study represents the largest and most culturally diverse study on math anxiety. Importantly, the analyses explicitly model the fact that math anxiety is accounted for by factors that occur at multiple nested levels of analysis—that students are nested within schools, which are themselves nested within countries.

The findings from this multilevel, cross-national study of math anxiety reveal that the strongest and most consistent predictors of mathematics anxiety can be found at the individual level. In other words, factors that are unique to individual students, independently of the country or educational environment that they are situated within, are the most consistent predictors of the level of math anxiety that they experience. The strongest predictor of student math anxiety at the individual level was the student’s perception of teacher competence (Δ= 0.274). While effects of the education environment level were less consistent, teacher confidence in teaching math was negatively but weakly associated with students’ math anxiety (Δ= 0.028). Furthermore, the frequency with which math homework was assigned within the education environment that students are in also weakly contributed to math anxiety (Δ= 0.010). Interestingly, the above reported analyses do not reveal any predictors of math anxiety at the country level.

With respect to the relationship between math anxiety and math achievement, the present analyses reveal a consistent relationship between individual math anxiety and math achievement across the globe (TIMSS Grade 4: average Δ= 0.531, TIMSS Grade 8: average Δ= 0.462, PISA: average Δ= 0.383). Moreover, this effect is nuanced by the fact that, at least in some countries, the average math anxiety experienced by students within the same educational context (i.e., school or classroom) is related to the individual students’ math achievement, independently of the individual level of math anxiety that students report (TIMSS Grade 4: average Δ= 0.382, TIMSS Grade 8: average Δ= 0.395, PISA: average Δ= 0.466). This means that when considering how math anxiety exerts its effect on math achievement, it is important to take into account the effects of the environment within which the student is learning math.

Importantly, we also found evidence of cross-level interactions—where the strength of the individual and contextual effect of math anxiety is dependent on variables attributable to the larger environment in which these effects are nested. First, we found evidence to suggest that teacher confidence in teaching math is associated with a smaller effect of an individual students’ math anxiety on their math achievement. Second, we found that in countries with higher levels of uncertainty avoidance, the effects of the education environment–average math anxiety on student math achievement are lower.

### Individual and Contextual Effect of Math Anxiety on Math Achievement.

While the current study employed databases that differ with respect to age groups, countries sampled, and method of measuring math anxiety and math achievement, there was, nevertheless, a surprising level of consistency in the results regarding the individual and contextual effect of math anxiety on math achievement. At the individual level, our results from all three databases suggest that students in virtually all countries exhibited a negative association between individual math anxiety and math achievement. Indeed, the observed average effect size of the individual-level effect across all three databases (TIMSS Grade 4: average Δ= 0.531, TIMSS Grade 8: average Δ= 0.462, PISA: average Δ= 0.383) is similar magnitude to that of the average effect size (*r* = −0.28, converted to d = 0.580) (45) as reported by Barroso et al. (3). At the education environment level, results suggest that approximately half the countries exhibited a statistically significant association between education environment–average math anxiety and math achievement (TIMSS Grade 4: average Δ= 0.382, TIMSS Grade 8: average Δ= 0.395, PISA: average Δ= 0.466).

The consistency of the individual effect of math anxiety across databases and countries is concordant with the idea that math anxiety is a “global phenomenon” (ref. 31, p. 52) and highlights the universality of the adverse effects of individual math anxiety on math performance. Further, we have revealed a contextual effect of math anxiety, whereby the average level of student math anxiety in one’s immediate education environment makes an independent contribution to explaining variability in math achievement. However, results suggest that the contextual effect is highly variable across countries, with only half of the sampled countries exhibiting a statistically significant contextual effect. This suggests there is heterogeneity in the mechanisms through which education environment–average math anxiety affects math achievement and suggests that studies examining the interactions between these variables may have low generalizability across countries.

### Individual and Environmental Predictors of Math Anxiety.

Our results indicate that math anxiety is associated with a variety of individual and environmental factors. Our findings reinforce research findings from multiple previous studies. For example, we found that there are gender differences in math anxiety (Δ= 0.099) and that students’ attitudes toward the learning environment are negatively associated with math anxiety (Δ= 0.100). These results reflect extant results suggesting that both student gender (42, 46, 47) and student attitudes (26, 44) are associated with math anxiety. Given that virtually all the independent variables at the individual level were associated, albeit weakly, with math anxiety, our findings support the notion that math anxiety is a multifaceted phenomenon.

At the education environment level, we found far fewer variables associated with math anxiety and generally, with much lower effect sizes. However, the variables that were found to be associated with math anxiety align with prior findings, providing an additional weight of evidence. Specifically, teacher’s confidence in math teaching—which is negatively correlated with teacher math anxiety (48, 49)—was found to be related to lower student math anxiety (Δ= 0.028), similar to previous findings (43, 50). Similarly, we found the frequency of homework to be associated with higher math anxiety (Δ= 0.010), which is consistent with prior literature (44, 51).

Taken together, our results consolidate current understanding of the causes of math anxiety and support the notion that math anxiety is affected by multiple factors. Further, our results also suggest that, in contrast to predictors at the individual level, correlates of math anxiety at the education environment level are more specific and are limited to only a few factors.

### Individual and Contextual Effects in the Context of Other Predictors.

When considering math anxiety as a predictor of math achievement in the context of other potential predictors of math achievement, we find that the average individual and contextual effects of math anxiety remain strong predictors of math achievement (individual effect: Δ= 0.476; contextual effect: Δ= 0.267). Interestingly, two cross-level interactions were found. First, higher teacher confidence was related with a weaker individual effect of math anxiety, and higher country uncertainty avoidance is associated with a lower contextual effect of math anxiety. While the effects are generally small, these results are preliminary evidence to suggest that variables at higher levels could not only simply predict student math anxiety but also, modulate the individual and contextual effects of math anxiety.

### Teachers Playing a Critical Role.

In the current study, we found evidence to suggest that teachers may play a central role in the effects of math anxiety. Specifically, we find 1) students’ perception of teacher competence to be the strongest predictor of student math anxiety at the individual level (Δ= 0.274), 2) negative associations between teacher confidence in teaching math and math anxiety (Δ= 0.028), and 3) teacher confidence in teaching math to also be associated with a weaker relationship between individual math anxiety and math achievement. These results suggest that both instruction quality and teacher affect may be related to student math anxiety.

A recent meta-analysis examining the link between teacher self-efficacy and instruction quality has suggested that the two are moderately related (*r* = 0.28) (52), with some researchers proposing that teacher self-efficacy predicts later instructional quality (e.g., ref. 53), while others suggest that instructional quality predicts later teacher self-efficacy (e.g., ref. 54). Together, these studies suggest that teacher self-efficacy and instructional quality, while related, are independent constructs. In this context, our results suggest that the improvement of both instructional quality and teacher confidence may be potential avenues of reducing student anxiety.

It is important to note, however, that while our results show a strong relation between student perception of teacher competence and math anxiety, it is unknown whether student perceptions would change, and to what degree, with improvements to instruction quality. Further, some extant studies suggest that teacher confidence and teacher math anxiety are negatively correlated (48, 49), and it is unclear whether the association between teacher confidence and math anxiety is due to this negative correlation. Finally, as our study utilized cross-sectional data, the casual direction between the variables would need to be experimentally and longitudinally confirmed. In sum, our results forward evidence suggesting the central role of the teacher in student math anxiety, but future experimental studies would be required to address the abovementioned issues.

### The Effects of Homework.

Interestingly, our results suggest that homework may play a significant role in math anxiety. We found that the frequency of homework assigned is related with higher math anxiety (Δ= 0.010), which is consistent with previous findings (44, 51). Similarly, we also found evidence to suggest that parental involvement with homework is associated with an increase in math anxiety (Δ= 0.126), which again is in line with previous findings (55). These findings suggest that homework and the degree to which parents are involved in their children’s homework must be considered carefully in any future study of math anxiety as well as potential interventions to alleviate math anxiety.

### Conclusion.

As a whole, the present data reveal that mathematical anxiety and its relationship to math achievement are affected by factors that are unique to the individual child, the educational context within which the children learn, and one’s country of residence. These data highlight the importance of moving beyond positioning math anxiety as something that exists only within an individual student but rather, positioning it as a construct that is affected, in complex ways, by factors that are nested within the educational environment and the country of the learner.

## Methods

### Data Sources—TIMSS Grade 4 and Grade 8 and PISA.

The current study draws upon the TIMSS 2015 Grade 4 and Grade 8 databases and the PISA 2012 database. While a more recent PISA database is available, the most contemporary database does not include the measurements of math anxiety. The sample design for the TIMSS and the PISA databases is a stratified two-stage random sample design (38, 39). Both studies draw a sample of schools from participating countries as a first stage. As a second stage, one intact class of students is selected from each of the sampled schools in the case of the TIMSS (38), and a random selection of eligible students who are not necessarily from the same class is drawn in the case of the PISA (39). Consequently, the second level of nesting can be most aptly described as the classroom level for the TIMSS and the school level for the PISA. For current purposes, we will refer to this second level as the education environment level. The differences in data collection methods causes the statistical interpretation of the results to be somewhat different between the databases (*SI Appendix* has more details and other statistical considerations). Final datasets are made available with the article (https://osf.io/825qm/).

### Participants.

Combining all three databases, the total sample size exceeded 1 million participants (*n* = 1,175,515). The final sample for the TIMSS Grade 4 database included 404,688 students (196,412 females; mean age = 10.11; SD = 0.61) from 21,600 classrooms (mean number of students per classroom = 18.74) across 54 countries (mean number of students per country = 7,494.22). The final sample for the TIMSS Grade 8 included 290,653 students (144,277 females; mean age = 14.23; SD = 0.79) from 14,426 classrooms (mean number of students per classroom = 20.15) across 46 countries (mean number of students per country = 6,318.54). The final sample for the PISA database included 480,174 students (242,375 females; mean age = 15.78; SD = 0.29) from 18,139 schools (mean number of students per school = 26.47) across 65 countries (mean number of students per country = 7,387.29). For both the TIMSS databases and the PISA database, a student questionnaire (containing questions regarding math anxiety) is administered immediately after math achievement assessments in the same session on the same day (38, 39).

### Description of Key Variables.

#### Math achievement.

Across all three databases, math achievement was measured through a comprehensive test, targeting a wide variety of mathematical skills and concepts. The TIMSS Grade 4 assessment measures topics related to number, geometry shapes and measure, and data display. The TIMSS Grade 8 assessment measures topics related to number, algebra, geometry, data, and chance (38). The PISA measures topics related to change and relationships, quantity, space and shape, and uncertainty and data (39). Studies that have compared math achievement assessed by the TIMSS and the PISA have found that questions in the TIMSS tend to be more theoretically oriented and that questions in the PISA tend to be more application oriented (56).

In both the TIMSS and the PISA, math achievement scores of students were measured using a rotating booklet design, whereby students complete only a subset of all assessment items (38, 39). As such, some degree of measurement error is introduced by this method of assessment (57). As a reflection of this uncertainty introduced by the rotating booklet design, five plausible values for each individual are provided by the TIMSS and PISA databases as a representation of proficiency. Correct analyses of plausible values require separate identical analyses for each plausible value with results integrated using principles from multiple imputation.

#### Math anxiety.

As part of all three achievement tests, students were asked to complete a series of self-report measures, including questions about math anxiety. On both the Grade 4 and Grade 8 TIMSS assessments, students were asked to indicate on a 4-point Likert scale (1 = agree a lot, 4 = disagree a lot) the extent to which they agreed with the statement “Mathematics makes me nervous.” For the PISA, math anxiety was operationalized as an observed variable calculated as the mean score of five individual test items. Students were asked to indicate on a 4-point Likert scale the extent to which they agreed with each of the following statements (1 = strongly agree, 4 = strongly disagree): “I often worry that it will be difficult for me in mathematics classes,” “I get very tense when I have to do mathematics homework,” “I get very nervous doing mathematics problems,” “I feel helpless when doing a mathematics problem,” and “I worry that I will get poor grades in maths.” All items were reverse coded where appropriate.

#### Predictors of math anxiety and math achievement.

Predictors of math anxiety were extracted from the TIMSS Grade 4 database. It was not possible to investigate the predictors of math anxiety in the other two databases due to unavailable data. Specifically, TIMSS Grade 8 did not collect any data regarding the students’ home and parents, and it is not possible to examine teacher’s effects in the PISA, as students are not necessarily from the same classroom.

The TIMSS Grade 4 database provides a rich set of potentially relevant variables that may affect math anxiety and math achievement, and the current study takes full advantage of this by including these variables as predictors of math anxiety and math achievement across three levels of analysis: individual level, education environment level, and country level.

At the individual level (L1), variables were subdivided into three categories: 1) student-specific factors, 2) past and present extracurricular mathematics training, and 3) parent and home factors. Student-specific factors included student self-report items related to student gender, student attitudes toward the math teacher, and student attitudes toward the school. Past and present extracurricular mathematics training included parent’s self-report items related to years of preschool education, home mathematics activities during preschool, and current extracurricular tutoring/lessons. Parent and home factors included parent’s self-report items related to parental involvement in mathematics homework, parental attitudes toward mathematics and science, parents’ highest education level, parents’ occupation, and home socioeconomic status.

At the classroom level (L2), variables were subdivided into two categories: 1) classroom factors and 2) teacher-specific factors. Classroom factors include weekly time spent on mathematics, frequency of mixed-ability grouping, frequency of same-ability groups, and math homework frequency. Teacher-specific factors include teacher gender, teacher satisfaction with work, teacher confidence in teaching mathematics, teacher years of teaching experience, and teacher major.

Finally, at the country level (L3), we included socioeconomic development and cultural dimensions from Hofstede et al. (58). Socioeconomic development was represented with the United Nations Human Development Index (59). Cultural differences between countries were represented by five dimensions (individualism–collectivism, power distance, uncertainty avoidance, masculinity, and long-term orientation) as proposed by Hofstede et al. (58).

The HDI is an index that accounts for multiple facets of human development in a specific country; this includes gross domestic product per capita, life expectancy, adult literacy rate, and school enrollment ratio. For the current study, we used the values from 2015, the year of data collection for the TIMSS Grade 4.

As reviewed in the Introduction, little is known about whether there are between-country differences in math anxiety and what country-level variables may motivate these differences. The current study included the five cultural dimensions of Hofstede et al. (58) as a preliminary exploration of the potential of between-country differences. Results from the current study may aid future studies in identifying specific math-related country factors, such as between-country differences in math education environment, cultural perceptions of math, or expectations placed on students regarding math achievement.

The individual–collectivism index describes the degree to which individuals in a culture are loosely associated with or tightly integrated into societal groups. The uncertainty avoidance index reflects the degree of cultural acceptance of uncertain or ambiguous situations and the degree to which members of a culture will try to avoid these situations. Cultures with high uncertainty avoidance are more likely to have well-defined rules of behaviors for interpersonal interactions. The power distance index reflects the acceptance and expectations of power inequality and authority of persons higher in hierarchical organizations. The masculinity index reflects the degree to which a culture’s dominant values are related to achievement and success (masculine) or related to caring for others and quality of life (feminine). The long-term orientation index describes the degree to which individuals in a culture are directed toward future rewards or the realization of present needs and desires.

All predictors were reverse coded and operationalized as latent variables where appropriate. The substantive basis for the included variables and details of the item wordings of main variables and covariates are in *SI Appendix*.

### Analyses.

Missing data were handled using multiple imputation (60, 61). Analyses were carried out using Mplus 8.3 with the maximum likelihood estimator with robust SEs (62). All continuous variables were standardized (mean = 0, SD = 1) prior to estimation to remove nonessential multicollinearity (63). For all models below, math anxiety as a predictor was grand-mean centered, and a manifest aggregation approach was used to estimate the contextual effect (64). This implies that the higher-level regression coefficients are a direct estimation of the contextual effect that controls for lower-level variations (65, 66). Effect sizes for L1 to L3 effects were calculated according to Marsh et al. (64) and Tymms (67):

$$\Delta =2\times \beta \times \sigma_{\mathrm{pred}}/\sigma_{y},$$

where β is the unstandardized regression coefficient, $\sigma_{\mathrm{pred}}$ is the SD of the predictor variable, and $\sigma_{y}$ is the SD of the outcome variable. This effect size metric is comparable with Cohen’s *d* (41).

Where applicable, goodness of fit of the models was assessed with the χ^2^ test statistic, CFI, TLI, and RMSEA. Typical cutoff scores for excellent and adequate fit are CFI and TLI > 0.95 and > 0.90, respectively, and RMSEA < 0.06 and < 0.08, respectively (68). More details regarding weighting, confirmatory factor analysis, and assessment of model fit are given in *SI Appendix*.

#### Is There a Contextual Effect of Math Anxiety at the Education Environment Level?

We first sought to establish a baseline model by employing a total group analysis using a two-level model for each database to account for the nesting of students in the immediate education environment. This baseline model will yield estimates of the L1 individual effect and ascertain whether there is evidence for an L2 contextual effect. Adopting the notation used by Raudenbush and Bryk (69), we have the following model:

$$\begin{array}{c} \text{L1}:\left(Y_{i,j,k}-\beta_{0,0,k}\right)=\pi_{0,j,k}+\pi_{1,j,k}\left(X_{i,j,k}-{\bar{X}}_{\cdot ,\cdot ,k}\right)+e_{i,j,k}\\ \text{L2}:(\pi_{0,j,k}-\beta_{0,0,k})=\beta_{0,1,k}\left(X_{\cdot ,j,k}-{\bar{X}}_{\cdot ,\cdot ,k}\right)+r_{0,j,k}, \end{array}$$

where the variable $Y_{i,j,k}$ is the math achievement for person *i* in education environment *j* in country *k*. The predictors individual math anxiety $\left(X_{i,j,k}\right)$ and education environment–average math anxiety $\left(X_{\cdot ,j,k}\right)$ are centered with respect to the country means $\left({\bar{X}}_{\cdot ,\cdot ,k}\right)$. To account for the fact that education environments are nested into countries, country membership was treated as a stratification variable; as such, SEs and test statistics were corrected for the nesting of education environment within the country. It is noted that since math achievement was centered around country means $\left(\beta_{0,0,k}\right)$, we have removed the between-country variation in math achievement that would have been otherwise attributed to L2 (70).

#### Are There Between-Country Differences in the Contextual Effect at the Education Environment Level?

Next, to ascertain whether the baseline model generalizes to all countries, a multigroup two-level model for each database was estimated. Specifically, we modeled the same L1 individual effect and L2 contextual effect as the previous analysis. Country membership was treated instead as a fixed effect grouping variable. To test for between-country differences in the magnitude of the individual and contextual effects, two multigroup two-level models were compared for each database: 1) an unconstrained model in which the structural parameters at L1 and L2 are allowed to vary across countries and 2) a constrained model in which the structural parameters at L1 and L2 are held constant across countries. A significant reduction in model fit when structural parameters are held constant across countries will suggest that there are significant between-country differences in the magnitude of the individual and contextual effects.

#### What Individual and Environmental Factors Predict Math Anxiety?

The following analyses was performed only on TIMSS Grade 4 due to the lack of available data in the other two databases. To examine whether the same individual and environmental factors may predict math anxiety, we estimated a three-level model with math anxiety as the outcome variable. Math anxiety is grand-mean centered. L1, L2, and L3 predictors were also added:

$$\begin{array}{c} \text{L1}:Y_{i,j,k}=\pi_{0,j,k}+\pi_{1,j,k}{\left(\text{STU}\;\text{GENDER}\right)}_{i,j,k}+\cdots +e_{i,j,k}\\ \text{L2}:\pi_{0,j,k}=\beta_{0,0,k}+\beta_{0,1,k}{\left(\text{TEA}\;\text{GENDER}\right)}_{.,j,k}+\cdots +r_{0,j,k}\\ \text{L3}:\beta_{0,0,k}=\gamma_{0,0,0}+\gamma_{0,0,1}{\left(\text{HDI}\right)}_{.,.,k}+\cdots +\mu_{0,0,k}. \end{array}$$

#### Are the Individual and Contextual Effects of Math Anxiety Robust to Other Predictors of Math Achievement?

The following analyses were performed only on TIMSS Grade 4 due to the lack of available data in the other two databases. A possible area of between-country differences in the relations between math anxiety and math achievement is between-country differences in the magnitude of the L1 individual effect and L2 contextual effect. To examine this, we estimated a three-level model with random slopes and included L1, L2, and L3 predictors into the model:

$$\begin{array}{c} \text{L1}:Y_{i,j,k}=\;\pi_{0,j,k}+\pi_{1,j,k}\left(X_{i,j,k}-{\bar{X}}_{\cdot ,\cdot ,\cdot }\right)+\pi_{2,j,k}{\left(\text{STU}\;\text{GENDER}\right)}_{i,j,k}+\cdots +e_{i,j,k}\\ \text{L2}:\pi_{0,j,k}=\beta_{0,0,k}+\beta_{0,1,k}\left(X_{\cdot ,j,k}-{\bar{X}}_{\cdot ,\cdot ,\cdot }\right)+\beta_{0,2,k}{\left(\text{TEA}\;\text{GENDER}\right)}_{.,j,k}+\cdots +r_{0,j,k}\\ \pi_{1,j,k}=\beta_{1,0,k}+\beta_{1,2,k}{\left(\text{TEA}\;\text{GENDER}\right)}_{.,j,k}+\cdots +r_{1,j,k}\\ \text{L3}:\beta_{0,0,k}=\gamma_{0,0,0}+\gamma_{0,0,1}\left(X_{\cdot ,\cdot ,k}-{\bar{X}}_{\cdot ,\cdot ,\cdot }\right)+\gamma_{0,0,2}{\left(\text{HDI}\right)}_{.,.,k}+\cdots +\mu_{0,0,k}\\ \beta_{0,1,k}=\gamma_{0,1,0}+\gamma_{0,1,1}{\left(\text{HDI}\right)}_{.,.,k}+\cdots +u_{0,1,k}\\ \beta_{1,0,k}=\gamma_{1,0,0}+\gamma_{1,0,1}{\left(\text{HDI}\right)}_{.,.,k}+\cdots +u_{1,0,k}. \end{array}$$

Math anxiety and individual and environmental predictors from the previous analysis were added as predictors of math achievement. In doing so, we control for the influences of these predictors and examine whether the variance explained by math anxiety overlaps with that accounted for by other predictors. The individual effect $\left(\pi_{1,j,k}\right)$ and contextual effect $\left(\beta_{0,1,k}\right)$ of math anxiety are modeled as random slopes, and we regressed the L2 and L3 random slopes onto the L2 and L3 predictors. This will allow us to examine whether these individual and environmental factors may account for the between-environment and between-country differences in the magnitude of effect of the individual and contextual effects.

## Supplementary Material

Supplementary File

## Data Availability

All study data are included in the article and/or *SI Appendix*.
