# Supplementary material for: Disentangling the individual and contextual effects of math anxiety: A global perspective
Source: Proc Natl Acad Sci U S A. 2022 Feb 7;119(7):e2115855119. doi: 10.1073/pnas.2115855119 (PMC8851526; doi:10.1073/pnas.2115855119)
Supplement: Supplementary File [file pnas.2115855119.sapp.pdf]

PNAS
www.pnas.org

Supplementary Information for
Disentangling the Individual and Contextual Effects of Math Anxiety: A Global Perspective

Nathan Lau, Zachary Hawes, Paul Tremblay & Daniel Ansari

Corresponding Author: Lau (tlau97@uwo.ca)

This PDF file includes:

- Supplementary text
- Tables S1 to S5
- SI References

Additional Statistical Considerations

Classroom and School Level Variations in the TIMSS and PISA

One major difference between the sampling methodologies of the TIMSS and PISA datasets is the primary sampling unit. While the primary sampling unit of the TIMSS dataset is the classroom while the primary sampling unit for the PISA is the school (1). As such, interpretations of level-2 variations for the TIMSS and PISA models would be different. Specifically, variations at level-2 can mostly be attributed to the classroom for the TIMSS and the school for the PISA.

The omitted levels for all three ML-SEMs would introduce imprecision in the estimated variations that exists at different levels. Simulation studies have shown the variance attributed to an omitted level is divided between the flanking levels (2). As such, the omission of the school level for the TIMSS would inflate variance attributed to the classroom and country levels, while the omission of the classroom level for the PISA would inflate variance attributed to the individual and school levels.

In both cases the omission of a level inflates variations attributed to level-2 in the ML-SEM modelled in the current study. While the statistically significant average contextual effect at level-2 in all three ML-SEMs suggests that the math anxiety level of students' immediate educational environment (i.e., the school and the classroom) have an addictive detrimental effect on students' math achievement, the specific level of the contextual effect (i.e., whether the effect is at the classroom level or school level or both) cannot be determined from the data. Whether the contextual effect exists on the classroom- or school-levels or both is a subject for future study.

Sampling Weights

Both the TIMSS and the PISA employ a stratified two-stage random sample design (1, 3). The usage of sampling weights is required to adjust for the non-independency introduced by the sampling design (4). For the TIMSS, sample weights for the student level were calculated as the product of WGTFAC3 and WGTADJ03, and sample weights for the classroom level were calculated as the product of WGTFAC01, WEGADJ01, WGTFAC02, and WTGADJ02 (5). For the PISA, sample weights for the student level was computed as the ratio between the final student

weight and the final school weight (i.e., W_FSTUWT/ W_FSCHWT) and sample weights for the school level was the final school weight (W_FSCHWT; refs. 1–3).

Measurement Model for the Latent Variables in TIMSS Grade 4

Multiple covariates of math anxiety and math achievement in the final model contained multiple items and were operationalized as latent variables. Specifically, latent variables at the student level were student attitudes towards the math teacher, student attitudes towards the school, current extracurricular tutoring/lessons, home math activities during preschool, and parental attitudes towards math and science. Latent variables at the teacher level were teacher confidence in teaching math and teacher satisfaction with work (see appendix B & C for item descriptions). The factor structure of these latent variables was examined with a measurement model CFA, one for each level. Results indicate that latent variables at both individual level, $\chi^2_{(650)}$ = 56529.33, p < .001, CFI = .990, TLI = .989, RMSEA = .015, SRMR = .045, and classroom level, $\chi^2_{(89)}$ = 592.77, p < .001, CFI = .977, TLI = .973, RMSEA = .004, SRMR = .028, were well fitting.

Model Fit

Model fit were reported where available. However, several models were saturated or contained random slopes. In both cases, conventional model fit indices were not available.

Variable Description

Table S1. Item description of variables.

**Variable**	**Item Description**
**Individual Level**	
Math Anxiety	TIMSS Grade 4:<br>*ASBM03E – Mathematics makes me nervous<br><br>TIMSS Grade 8:<br>*BSBM19E – Mathematics makes me nervous<br><br>PISA:<br>* ST42Q01 – I often worry that it will be difficult for me in mathematics classes<br>* ST42Q03 – I get very tense when I have to do mathematics homework<br>* ST42Q05 – I get very nervous doing mathematics problems<br>* ST42Q08 – I feel helpless when doing a mathematics problem
Student Gender	ASBG01 - Are you a girl or a boy?<br><br>1) Girl, 2) Boy
Student Attitudes towards the Math Teacher	*ASBM02A – I know what my teacher expects me to do<br>*ASBM02B – My teacher is easy to understand<br>*ASBM02C – I am interested in what my teacher says<br>*ASBM02D – My teacher gives me interesting things to do<br>*ASBM02E – My teacher has clear answers to my questions<br>*ASBM02F – My teacher is good at explaining mathematics<br>*ASBM02G – My teacher lets me show what I have learned<br>*ASBM02H – My teacher does a variety of things to help us learn<br>*ASBM02I – My teacher tells me how to do better when I make a mistake<br>*ASBM02J – My teacher listens to what I have to say

Student Attitudes towards the School	*ASBG11A – I like being in school<br>*ASBG11B – I feel safe when I am at school<br>*ASBG11C – I feel like I belong at this school<br>*ASBG11D – I like to see my classmates at school<br>*ASBG11E – Teachers at my school are fair to me<br>*ASBG11F – I am proud to go to this school<br>*ASBG11G – I learn a lot in school
Years of Preschool Education	ASBH05B - Approximately, how long was your child in these programs [referring to pre-primary education] altogether?<br><br>1) Did not attend, 2) Less than 1 year, 3) 1 year, 4) 2 years, 5) 3 years, 6) 4 years or more
Home Math Activities during Preschool	Before your child began primary/elementary school, how often did you or someone else in your home do the following activities with him or her?<br><br>*ASBH02J – Say counting rhymes or sing counting songs<br>*ASBH02K – Play with number toys (e.g., blocks with numbers)<br>*ASBH02L – Count different things<br>*ASBH02M – Play games involving shapes (e.g., shape sorting toys, puzzles)<br>*ASBH02N – Play with building blocks or construction toys<br>*ASBH02O – Play board or card games<br>*ASBH02P – Write numbers<br><br>1) Often, 2) Sometimes, 3) Never or almost never
Current Extracurricular Tutoring/Lessons	ASBH10BA – For how many of the last 12 months has your child attended extra lessons or tutoring?<br><br>1) Did not attend, 2) Less than 4 months, 3) 4-8 months, 4) More than 8 months

Parental Involvement in Homework	How often do you or someone else in your home do the following things?<br><br>*ASBH09BB –Help your child with homework<br>*ASBH09BC –Review your child's homework to make sure it is correct<br><br>1) Every day, 2) 3 or 4 times a week, 3) 1 or 2 times a week, 4) Less than once a week, 5) Never or almost never
Parental Attitudes towards Math	How much do you agree with these statements about mathematics and science?<br>ASBH16A – Most occupations need skills in math, science, or technology<br>ASBH16D – My child needs mathematics to get ahead in the world<br>ASBH16G – Mathematics is applicable to real life<br>ASBH16H – Engineering is necessary to design things that are safe and useful
Parent’s Highest Education Level	ASDHEDUP – Highest level of education of either parent<br><br>1) Finished some primary or lower secondary or did not go to school, 2) Finished lower secondary, 3) Finished upper secondary, 4) Finished post-secondary education, 5) Finished university or higher
Parent’s Occupation	*ASDHOCCP – Highest level of occupation of either parent<br><br>1) Professional, 2) Small Business Owner, 3) Clerical, 4) Skilled Worker, 5) General Laborer, 6) Never worked outside home, 7) Not Applicable [7 recoded as missing]

Home Socioeconomic Status	ASBG04 – Number of books in the home:<br>1) 0-10, 2) 11-25, 3) 26-100, 4) 101-200, 5) More than 200
**Classroom Level**	
Weekly Time Spent on Math	ATBM01 – In a typical week, how much time do you spend teaching mathematics to the students in this class? (minutes)<br><br>Minutes in Integer
Frequency of Mix-Ability Grouping	ATBM03H – In teaching mathematics to this class, how often do you ask students to do the following? Work in mixed ability groups<br><br>1) Every or almost every lesson, 2) About half the lessons, 3) Some lessons, 4) Never
Frequency of Same-Ability Grouping	ATBM03I – In teaching mathematics to this class, how often do you ask students to do the following? Work in same ability groups<br><br>1) Every or almost every lesson, 2) About half the lessons, 3) Some lessons, 4) Never
Homework Frequency	ATBM07B – When you assign mathematics homework to the students in this class, about how many minutes do you usually assign? (Consider the time it would take an average student in your class.)<br><br>1) 15 minutes or less, 2) 16-30 minutes, 3) 31-60 minutes, 4) more than 60 minutes
Teacher Gender	ATBG02 – Are you female or male?<br><br>1) Female, 2) Male
Teacher Satisfaction with Work	How often do you feel the following way about being a teacher?

	*ATBG10A – I am content with my profession as a teacher<br>*ATBG10B –I am satisfied with being a teacher at this school<br>*ATBG10C – I find my work full of meaning and purpose<br>*ATBG10D – I am enthusiastic about my job<br>*ATBG10E – My work inspires me<br>*ATBG10F – I am proud of the work I do<br>*ATBG10G – I am going to continue teaching for as long as I can<br><br>1) Very often, 2) Often, 3) Sometimes, 4) Never or almost never
Teacher Confidence in Teaching Math	In teaching mathematics to this class, how would you characterize your confidence in doing the following?<br><br>*ATBM02A – Inspiring students to learn mathematics<br>*ATBM02B – Showing students a variety of problem solving strategies<br>*ATBM02C – Providing challenging tasks for the highest achieving students<br>*ATBM02D – Adapting my teaching to engage students' interest<br>*ATBM02E – Helping students appreciate the value of learning mathematics<br>*ATBM02F – Assessing student comprehension of mathematics<br>*ATBM02G – Improving the understanding of struggling students<br>*ATBM02H – Making mathematics relevant to students<br>*ATBM02I – Developing students' higher-order thinking skills<br><br>1) Very high, 2) High, 3) Medium, 4) Low
Teacher Years of Teaching Experience	ATBG01 – By the end of this school year, how many years will you have been teaching altogether?<br><br>Integer response.

Teacher Years of Formal Education	ATBG04 – What is the highest level of formal education you have completed?<br><br>1) Did not complete <Upper secondary education—ISCED Level 3>,<br>2) <Upper secondary education— ISCED Level 3>,<br>3) <Post-secondary, non-tertiary education—ISCED Level 4>,<br>4) <Short-cycle tertiary education—ISCED Level 5>,<br>5) <Bachelor's or equivalent level—ISCED Level 6>,<br>6) <Master's or equivalent level—ISCED Level 7>,<br>7) <Doctor or equivalent level—ISCED Level 8>
Teacher Major	ATDM05 –Teachers Majored in Education and Mathematics [Derived Variable]<br><br>1) Major in primary education and major (or specialization) in mathematics,<br>2) Major in primary education but no major (or specialization) in mathematics,<br>3) Major in mathematics but no major in primary education,<br>4) All other majors<br>5) No formal education beyond upper-secondary<br>[Recoded into binary variable, 1 being major in education or mathematics, 0 being did not major in education or mathematics]
**Country-Level**	
Human Development Index	The human development index is a composite score of a country's development measured across three broad dimensions: long and healthy life, knowledge, and a decent standard of living. The health dimension is assessed by the life expectancy a typical citizen at birth. The education dimension is measured by the average years of schooling for adults above the age of 25, and the expected years of schooling for children at the first year of

	schooling. The standard living dimension is measured by the gross national income. The three dimensions are combined into a composite score by using geometric mean (8).
Individualism-Collectivism Index	Individual-Collectivism is a bipolar variable that refers the degree to which ties between individuals are loose (individualistic) or are tight (collectivist). An example question is, "Try to think of those factors that would be important to you in an ideal job; disregard the extent to which they are contained in your present job. How important is it to you to have sufficient time for your personal or family life?" (from 1 "of utmost importance to me" to 5 "of very little or no importance"). High values of individualism-collectivism index indicate higher individualism, and lower values indicate higher collectivism. Example taken from Hofstede et al. (9).
Power Distance Index	Power distance refers to the degree to which the less powerful members of an organization expect and accept that power is distributed unequally. An example question is, "How frequently, in your experience, does the following problem occur: employees being afraid to express disagreement with their managers?" (from "very frequently" to "very seldom"). High values of power distance index indicate less acceptance of inequalities with a country. Example taken from Hofstede et al. (9).
Uncertainty Avoidance Index	Uncertainty avoidance refers to the degree to which the members of a culture feel threatened by ambiguous or unknown situations. Societies with high uncertainty avoidance are less tolerant of ambiguous situations and are more likely to have well-defined rules for behaviors for social interactions. Societies with low uncertainty avoidance are more tolerant of ambiguous situations and have less strict or defined rules for behaviors for social interactions. An example question is, "Company rules should not be broken—even when the employee thinks it is in the company's

	best interest" (from 1 "I always feel this way" to 5 "I never feel this way.") High values of uncertainty avoidance indicate less acceptance of uncertainties. Example taken from Hofstede et al. (9).
Masculinity Index	Masculinity is a bipolar variable that refers to degree to which emotional roles are clearly distinct. Multiple masculine and feminine values are given in Hofstede et al. (9). The following statements are most pertinent to the classroom. Feminine values include: "average student is the norm; praise for weak students," "jealousy of those wo try to excel," "failing in school is a minor incident, "students underrate their own performance: ego-effacement," and "friendliness in teachers is appreciated." Masculine values include: "best student is the norm; praise for excellent students," "competition in class; trying to excel," "failing in school is a disaster," "students overrate their own performance: ego-boosting," and "brilliance in teachers is admired." Higher values of masculinity indicate higher degree of endorsement of masculine values, while lower values of masculinity indicate higher degree of endorsement of feminine values.
Long-Term Orientation Index	Long-term orientation is a bipolar variable that refers to the degree to which members of a culture are oriented towards future or to the past and present. Particularly, long-term oriented cultures endorse perseverance and thrift, while short-term oriented cultures endorse respect for tradition and the preservation of "face." Higher values of long-term orientation indicate a higher level of orientating towards the future, while lower values of long-term orientation indicate a higher degree of orienting towards the past and present.

* reverse coded. All items correspond to a 4-point Likert scale with 1 being agree a lot and 4 being disagree a lot unless otherwise specified. All items are found in the TIMSS Grade 4 unless otherwise specified. Binary categorical variables are recoded to be 0 and 1.

Substantive Basis for the Included Variables for TIMSS Grade 4

Individual Level Variables

Student and Teacher Gender

Multiple studies have found that adult females generally experience higher levels of math anxiety (10–14). However, evidence is more mixed in studies with children. While some studies have reported no gender difference in math anxiety (15–17) other studies have found that, similar to adults, girls experience higher levels of anxiety (18–20). Interestingly, there may be an interaction between a child's gender and the teacher's gender. For instance, Beilock and colleagues (21) have found gender specific effects of math anxiety whereby it is female students of math anxious female teachers to be more likely to be behind in math (21). However, a larger replication study have seem to suggest that both genders may experience lower math performance (22).

Student Attitudes

Extant research has revealed that student attitudes are strong predictors of math anxiety. For instance, students' own beliefs regarding their own math ability has been shown to be inversely associated with math anxiety (23, 24). Similarly, students' positive beliefs regarding the teacher efficacy and the school learning environment atmosphere have been shown to be negatively associated with math anxiety (25, 26). A more positive opinion regarding the teacher and learning environment may be associated with reduced math anxiety because stronger support from teachers and peers may help reduce negative expectations that could induce math anxiety.

Years in Pre-primary Education and Home Math Activities During Preschool

Not much is known regarding whether preschool attendance would have a positive or negative effect on math anxiety. Multiple studies have found that preschool teachers may exhibit math anxiety (27) and teachers' anxiety may be transmitted to students (21). Indeed, there is some support for the notion that children at that age already experience math anxiety and that it affects their math achievement (28, 29). However, few research studies have examined whether the attendance of pre-primary education would yield a net positive or negative effect on math anxiety when compared with not attending pre-primary education. On the one hand, earlier exposure to possible instigators of math anxiety would have one predict an association between pre-primary education and math anxiety. Specifically, one would predict that more years of pre-primary education would lead to more math anxiety. On the other hand, pre-primary education may endow

a protective effect by better equipping students for learning of formal mathematical concepts in the first grade, which suggests the more pre-primary education, the less math anxiety students will feel.

Similarly, home math activities during preschool may be related to math anxiety as it may endow a protective effect by preparing students for learning mathematics in the first grade. Interestingly, evidence is mixed, some studies have found home activities seem to reduce math anxiety (30), with other studies finding that home activities increases math anxiety (31)**.** However, extant studies tend to measure home math activities concurrent with the measurement of math anxiety. To the best of our ability, we have not found studies that examine how home math activities prior to formal schooling would affect later math anxiety.

Parental Involvement with Homework and Current Extracurricular Tutoring/Lesson

Surprisingly, extant research examining homework have generally found a negative effect of the amount of homework on math anxiety. For instance, a higher proportion of total homework time spent doing math homework is associated with higher math anxiety (26, 32). It is uncertain whether this may a reflect of children experiencing more difficulty with doing more math homework or have a higher quantity of math homework. Further, there is evidence to suggest that parents with high math anxiety who also help their children with homework seem to increase their children's math anxiety (33). Taken together, math homework can be an indicator of student who are already struggling or can be considered a vehicle through which parental math anxiety may be transmitted to students.

In contrast, few research studies have examined the effects of extra-curricular tutoring on math anxiety. Existing studies have designed and implemented intensive and specialized math tutoring session to general positive effect (34)**.** However, the effects of normal extracurricular tutoring have not been examined. The effects of extra-curricular tutoring may be complicated due to its relationship with student SES – that is, those students with higher SES may have a higher likelihood of receiving extra-curricular tutoring. However, similar with homework, extra-curricular tutoring may be an indicator of students who are already struggling. Taken together, one may expect a negative relationship between extra-curricular tutoring and math anxiety, but a positive relationship once SES is statistically accounted.

Parental Attitudes towards Math and Science

Parents serve as role models and influences children's perception and attitudes towards math. For instance, children of more math anxious teachers tend to endorse gender stereotypes in mathematics (21), and parental support and encouragement is positively related with student attitudes towards achievement in school (35, 36). Indeed, there is evidence to suggest that parental attitudes regarding the importance of mathematics seem to be associated with lowered math anxiety (37). Taken together, we should expect positive parental attitudes towards math and science to be related with lowered math anxiety.

Parents' Highest Education, Occupation and Home Socioeconomic Status

The evidence for the effects of socioeconomic status on math anxiety is somewhat mixed with some research suggesting a negative relationship (38–40) and other studies finding no relationship (26). Further, research has found that parental education is inversely related to math anxiety (41) and parental education can be considered a stable indicator of home SES (42). Taken together, evidence suggest that SES may be inversely related with student math anxiety, but some studies failed to find such a relation.

Classroom Level Variables

Frequency of Mixed-Ability and Same-Ability Grouping in the Classroom

To the best of our ability, we could not find extant studies that examine whether mixed- or same-ability groupings in the classroom would affect student math anxiety. However, research that examine the relationship between ability grouping and student attitudes seem to suggest that mixed ability grouping generally lead to more student positive outlook on math. In contrast, same-ability grouping seems to lead to more negative outlook on math (43, 44). This suggests the ability of one's peers may affect student attitudes towards mathematics. In light of this, it is quite plausible to expect ability grouping may affect math anxiety. For instance, one may expect that since lower ability children generally experience more math anxiety, same ability grouping may exacerbate the experience of math anxiety due to children being exposed to peers who are similarly anxious about math.

Weekly Time Spent on Math and Homework Frequency

See Parental Involvement with Homework.

Teacher Gender

See student gender.

Teacher Confidence in Teaching Math

A multitude of extant studies have examined how teachers may influence student math anxiety. For example, a number of studies have shown that teachers often experience math anxiety and that students with teachers who experience math anxiety may have lower math achievement (45, 46).

While the TIMSS Grade 4 do not include measurement of teacher anxiety, teacher confidence in teaching math is included. As previous research has indicated that teacher math anxiety is a strong negative predictor of teacher confidence in teaching math (47, 48), it is reasonable to hypothesize that teacher confidence may also be related with student math anxiety and achievement.

Teacher Years of Teaching Experience, Teacher Years of Formal Education, Teacher Major, and Teacher Satisfaction with Work

One important predictor of student math anxiety is classroom atmosphere (26). For instance, it has been suggested that certain pedagogical practice, such as, assuming many parts of the mathematical procedures to be simple and self-explanatory, using unique vocabulary without explanation, and overusing rote drills, may lower student confidence and increase student math anxiety (49). Indeed, negative experience with the teacher seems to be prominent way through which students acquire math anxiety (50).

While pedagogical practice differs between teachers, years of teaching experience, formal education, major, and satisfaction with work may serve as potential indicators for a teacher's ability to inspire a positive atmosphere in the classroom.

Country-Level Variables

Human Development Index and Cultural Dimensions

It has been previously observed that different countries vary widely in terms of the average student math anxiety, and it has bee postulated that cultural context may play a role in this between-country difference (51). Specifically, it has been proposed that between country differences in the parents' role in a child's mathematics education, and differences in competitiveness in the education environment may contribute to the observed differences (51). To control for between country differences in socio-economic status, the UN human development index is used. To control for between country differences in the behaviors of parents and teachers, Hofstede et al. (9)'s cultural dimensions are used.

Standardized Coefficient and Effect Sizes

Table S2 - Standardized Coefficient and Effect Sizes PISA

Country	Individual Effect – Coefficient	Individual Effect – Effect Size	Contextual Effect – Coefficient	Contextual Effect – Effect Size
Albania	-0.005(0.022)	-0.01(0.045)	0.022(0.034)	0.084(0.136)
Argentina	-0.114(0.016)***	-0.302(0.043)***	-0.118(0.072)	-0.385(0.215)
Australia	-0.213(0.011)***	-0.503(0.024)***	-0.11(0.038)**	-0.282(0.099)**
Austria	-0.176(0.013)***	-0.484(0.036)***	-0.137(0.074)	-0.549(0.264)*
Belgium	-0.115(0.01)***	-0.338(0.029)***	-0.138(0.065)*	-0.47(0.212)*
Brazil	-0.121(0.011)***	-0.342(0.031)***	-0.098(0.048)*	-0.356(0.163)*
Bulgaria	-0.128(0.015)***	-0.335(0.039)***	-0.189(0.057)**	-0.635(0.159)***
Canada	-0.27(0.016)***	-0.608(0.037)***	-0.058(0.033)	-0.193(0.113)
Chile	-0.132(0.013)***	-0.369(0.036)***	0.009(0.04)	0.06(0.26)
Colombia	-0.133(0.016)***	-0.347(0.04)***	-0.03(0.065)	-0.103(0.221)
Costa Rica	-0.142(0.017)***	-0.393(0.045)***	-0.115(0.094)	-0.351(0.274)
Croatia	-0.187(0.016)***	-0.471(0.038)***	-0.204(0.063)**	-0.56(0.171)**
Czechia	-0.206(0.013)***	-0.547(0.044)***	-0.189(0.086)*	-0.628(0.281)*
Denmark	-0.3(0.016)***	-0.648(0.035)***	-0.132(0.063)*	-0.414(0.173)*
Estonia	-0.267(0.016)***	-0.585(0.033)***	-0.059(0.037)	-0.179(0.111)
Finland	-0.227(0.017)***	-0.474(0.04)***	-0.171(0.109)	-0.543(0.349)
France	-0.13(0.016)***	-0.378(0.045)***	-0.044(0.043)	-0.155(0.147)
Germany	-0.161(0.013)***	-0.458(0.041)***	-0.221(0.064)**	-0.715(0.193)***
Greece	-0.203(0.016)***	-0.49(0.034)***	-0.165(0.048)**	-0.521(0.142)***
Hong Kong	-0.17(0.013)***	-0.405(0.036)***	-0.314(0.066)***	-0.852(0.222)***
Hungary	-0.133(0.015)***	-0.396(0.047)***	-0.174(0.053)**	-0.805(0.184)***
Iceland	-0.27(0.016)***	-0.569(0.031)***	-0.072(0.033)*	-0.189(0.087)*
Indonesia	-0.058(0.013)***	-0.16(0.034)***	-0.043(0.047)	-0.147(0.158)
Ireland	-0.213(0.017)***	-0.474(0.037)***	-0.098(0.04)*	-0.222(0.092)*
Israel	-0.109(0.015)***	-0.282(0.039)***	-0.186(0.081)*	-0.539(0.228)*
Italy	-0.148(0.008)***	-0.422(0.023)***	-0.042(0.051)	-0.16(0.192)
Japan	-0.121(0.013)***	-0.356(0.036)***	0.036(0.087)	0.133(0.301)
Jordan	-0.105(0.02)***	-0.259(0.048)***	-0.152(0.056)**	-0.388(0.141)**
Kazakhstan	-0.131(0.017)***	-0.338(0.042)***	0.021(0.055)	0.076(0.195)
Korea, Republic of	-0.091(0.031)**	-0.226(0.081)**	-0.108(0.093)	-0.274(0.221)
Latvia	-0.218(0.018)***	-0.496(0.04)***	-0.044(0.041)	-0.149(0.138)

Lithuania	-0.224(0.016)***	-0.523(0.039)***	-0.113(0.047)*	-0.332(0.127)**
Luxembourg	-0.177(0.011)***	-0.413(0.022)***	-0.179(0.077)*	-0.508(0.184)**
Macao	-0.192(0.014)***	-0.451(0.037)***	-0.07(0.097)	-0.175(0.237)
Malaysia	-0.109(0.02)***	-0.269(0.047)***	-0.081(0.06)	-0.201(0.147)
Mexico	-0.164(0.01)***	-0.409(0.023)***	-0.021(0.044)	-0.074(0.155)
Montenegro	-0.163(0.016)***	-0.367(0.039)***	-0.212(0.067)**	-0.795(0.218)***
Netherlands	-0.097(0.015)***	-0.318(0.055)***	-0.23(0.085)**	-0.763(0.258)**
New Zealand	-0.195(0.02)***	-0.444(0.048)***	-0.16(0.051)**	-0.446(0.141)**
Norway	-0.3(0.018)***	-0.635(0.047)***	-0.189(0.071)**	-0.588(0.225)**
Perm(Russian Federation)	-0.189(0.027)***	-0.448(0.063)***	-0.08(0.052)	-0.328(0.189)
Peru	-0.115(0.013)***	-0.318(0.036)***	-0.087(0.063)	-0.295(0.205)
Poland	-0.327(0.015)***	-0.71(0.041)***	-0.185(0.044)***	-0.642(0.217)**
Portugal	-0.159(0.014)***	-0.386(0.033)***	-0.105(0.05)*	-0.28(0.138)*
Qatar	-0.132(0.01)***	-0.342(0.026)***	-0.169(0.037)***	-0.695(0.113)***
Romania	-0.118(0.016)***	-0.286(0.049)***	-0.305(0.115)**	-1.016(0.295)**
Russian Federation	-0.211(0.015)***	-0.492(0.034)***	-0.075(0.032)*	-0.258(0.112)*
Serbia	-0.147(0.019)***	-0.38(0.059)***	-0.228(0.161)	-0.629(0.466)
Shanghai-China	-0.183(0.015)***	-0.453(0.036)***	-0.147(0.062)*	-0.365(0.151)*
Singapore	-0.166(0.015)***	-0.381(0.033)***	-0.321(0.046)***	-0.736(0.086)***
Slovakia	-0.208(0.016)***	-0.545(0.041)***	-0.135(0.053)*	-0.417(0.142)**
Slovenia	-0.139(0.011)***	-0.429(0.032)***	0.011(0.039)	0.068(0.24)
Spain	-0.169(0.011)***	-0.376(0.025)***	-0.068(0.023)**	-0.173(0.058)**
Sweden	-0.239(0.016)***	-0.521(0.034)***	0.036(0.073)	0.136(0.278)
Switzerland	-0.187(0.013)***	-0.449(0.03)***	-0.054(0.051)	-0.162(0.144)
Taiwan	-0.124(0.019)***	-0.312(0.05)***	0.052(0.141)	0.141(0.377)
Thailand	-0.053(0.018)**	-0.144(0.05)**	-0.132(0.09)	-0.508(0.338)
Tunisia	-0.033(0.017)*	-0.086(0.044)*	-0.121(0.12)	-0.348(0.434)
Turkey	-0.078(0.017)***	-0.24(0.054)***	-0.218(0.065)**	-0.935(0.242)***
United Arab Emirates	-0.163(0.01)***	-0.404(0.031)***	-0.248(0.071)**	-0.823(0.138)***
United Kingdom	-0.204(0.016)***	-0.472(0.038)***	-0.274(0.035)***	-0.666(0.105)***
United States	-0.259(0.018)***	-0.585(0.044)***	-0.161(0.082)*	-0.528(0.223)*
Uruguay	-0.161(0.016)***	-0.42(0.044)***	-0.162(0.083)	-0.533(0.279)
Viet Nam	-0.115(0.017)***	-0.339(0.052)***	-0.225(0.102)*	-0.696(0.277)*

Note. $^{***}p < 0.001$, $^{**}p < 0.01$, $^{*}p < 0.05$.

Table S3- Standardized Coefficient and Effect Sizes TIMSS 4

Country	Individual Effect – Coefficient	Individual Effect – Effect Size	Contextual Effect – Coefficient	Contextual Effect – Effect Size
Argentina, Buenos Aires	-0.179(0.022)***	-0.436(0.054)***	-0.122(0.05)*	-0.31(0.123)*
Armenia	-0.246(0.014)***	-0.546(0.03)***	-0.156(0.027)***	-0.356(0.064)***
Australia	-0.236(0.017)***	-0.542(0.039)***	-0.127(0.024)***	-0.465(0.087)***
Bahrain	-0.21(0.013)***	-0.474(0.028)***	-0.147(0.021)***	-0.348(0.05)***
Belgium (Flemish)	-0.178(0.017)***	-0.402(0.038)***	-0.046(0.035)	-0.107(0.082)
Bulgaria	-0.197(0.016)***	-0.483(0.038)***	-0.241(0.046)***	-0.628(0.11)***
Canada	-0.252(0.009)***	-0.584(0.021)***	-0.131(0.027)***	-0.359(0.073)***
Canada (Ontario)	-0.266(0.014)***	-0.593(0.03)***	-0.097(0.031)**	-0.261(0.083)**
Canada (Quebec)	-0.264(0.019)***	-0.582(0.041)***	-0.047(0.037)	-0.124(0.096)
Chile	-0.214(0.012)***	-0.485(0.027)***	-0.26(0.034)***	-0.716(0.071)***
Croatia	-0.266(0.024)***	-0.567(0.049)***	0.021(0.054)	0.048(0.125)
Cyprus	-0.284(0.013)***	-0.6(0.027)***	-0.024(0.02)	-0.056(0.046)
Czechia	-0.301(0.014)***	-0.659(0.028)***	0.018(0.037)	0.049(0.102)
Denmark	-0.231(0.019)***	-0.51(0.04)***	-0.113(0.027)***	-0.267(0.064)***
England	-0.2(0.015)***	-0.473(0.036)***	-0.095(0.034)**	-0.268(0.097)**
Finland	-0.216(0.016)***	-0.458(0.035)***	-0.11(0.027)***	-0.352(0.086)***
France	-0.218(0.015)***	-0.494(0.034)***	-0.07(0.038)	-0.189(0.101)
Georgia	-0.231(0.018)***	-0.532(0.041)***	-0.226(0.051)***	-0.583(0.128)***
Germany	-0.252(0.015)***	-0.553(0.033)***	-0.055(0.039)	-0.138(0.097)
Hong Kong	-0.237(0.018)***	-0.559(0.037)***	-0.199(0.036)***	-0.482(0.085)***
Hungary	-0.208(0.015)***	-0.504(0.036)***	-0.159(0.041)***	-0.431(0.117)***
Indonesia	-0.184(0.017)***	-0.471(0.047)***	-0.235(0.035)***	-0.644(0.086)***
Iran, Islamic Republic of	-0.285(0.021)***	-0.695(0.049)***	-0.117(0.049)*	-0.325(0.132)*
Ireland	-0.309(0.018)***	-0.658(0.037)***	0(0.039)	0.001(0.104)
Italy	-0.18(0.018)***	-0.398(0.039)***	0.036(0.036)	0.08(0.081)
Japan	-0.227(0.016)***	-0.469(0.032)***	-0.013(0.019)	-0.03(0.044)
Kazakhstan	-0.122(0.015)***	-0.354(0.045)***	-0.198(0.049)***	-0.608(0.142)***
Korea, Republic of	-0.171(0.014)***	-0.367(0.03)***	-0.051(0.04)	-0.122(0.097)
Kuwait	-0.182(0.023)***	-0.434(0.054)***	-0.063(0.026)*	-0.154(0.062)*
Lithuania	-0.241(0.018)***	-0.554(0.039)***	-0.123(0.041)**	-0.341(0.112)**
Morocco	-0.186(0.011)***	-0.488(0.029)***	-0.12(0.036)**	-0.35(0.102)**

Netherlands	-0.216(0.018)***	-0.456(0.038)***	-0.084(0.035)*	-0.188(0.078)*
New Zealand	-0.204(0.013)***	-0.483(0.03)***	-0.117(0.026)***	-0.336(0.072)***
Northern Ireland	-0.296(0.021)***	-0.644(0.043)***	-0.131(0.038)**	-0.316(0.093)**
Norway	-0.253(0.016)***	-0.535(0.032)***	-0.03(0.027)	-0.065(0.059)
Norway (4)	-0.257(0.016)***	-0.547(0.036)***	-0.09(0.027)**	-0.198(0.06)**
Oman	-0.197(0.011)***	-0.443(0.024)***	-0.125(0.024)***	-0.291(0.056)***
Poland	-0.271(0.015)***	-0.587(0.031)***	0.007(0.021)	0.017(0.048)
Portugal	-0.281(0.015)***	-0.632(0.032)***	-0.12(0.034)***	-0.286(0.081)***
Qatar	-0.184(0.013)***	-0.461(0.033)***	-0.152(0.034)***	-0.415(0.092)***
Russian Federation	-0.24(0.017)***	-0.597(0.041)***	-0.176(0.043)***	-0.475(0.1)***
Saudi Arabia	-0.161(0.012)***	-0.392(0.031)***	-0.208(0.039)***	-0.534(0.095)***
Serbia	-0.283(0.02)***	-0.599(0.042)***	0.2(0.042)***	0.495(0.107)***
Singapore	-0.133(0.008)***	-0.351(0.022)***	-0.427(0.03)***	-1.127(0.06)***
Slovakia	-0.255(0.014)***	-0.622(0.032)***	-0.035(0.045)	-0.092(0.119)
Slovenia	-0.262(0.018)***	-0.544(0.038)***	0.022(0.03)	0.05(0.069)
Spain	-0.282(0.013)***	-0.629(0.027)***	-0.035(0.026)	-0.09(0.068)
Sweden	-0.211(0.018)***	-0.469(0.041)***	-0.155(0.034)***	-0.379(0.083)***
Taiwan	-0.261(0.014)***	-0.544(0.028)***	-0.06(0.029)*	-0.146(0.076)
Turkey	-0.296(0.012)***	-0.698(0.032)***	-0.298(0.039)***	-0.738(0.083)***
United Arab Emirates	-0.15(0.006)***	-0.406(0.018)***	-0.294(0.022)***	-0.816(0.053)***
United Arab Emirates (Abu Dhabi)	-0.153(0.01)***	-0.397(0.03)***	-0.352(0.038)***	-0.974(0.086)***
United Arab Emirates (Dubai)	-0.152(0.008)***	-0.412(0.022)***	-0.235(0.035)***	-0.648(0.085)***
United States	-0.226(0.009)***	-0.544(0.023)***	-0.216(0.03)***	-0.554(0.067)***

Note. $^{***}p < 0.001$, $^{**}p < 0.01$, $^{*}p < 0.05$.

Table S4- Standardized Coefficient and Effect Sizes TIMSS 8

Country	Individual Effect – Coefficient	Individual Effect – Effect Size	Contextual Effect – Coefficient	Contextual Effect – Effect Size
Argentina, Buenos Aires	-0.166(0.017)***	-0.436(0.044)***	-0.046(0.065)	-0.127(0.179)
Armenia	-0.168(0.017)***	-0.377(0.037)***	0.041(0.055)	0.11(0.153)
Australia	-0.183(0.012)***	-0.521(0.034)***	-0.117(0.025)***	-0.537(0.11)***
Bahrain	-0.159(0.02)***	-0.369(0.045)***	-0.081(0.056)	-0.215(0.147)
Botswana	-0.114(0.015)***	-0.247(0.033)***	-0.116(0.032)***	-0.258(0.07)***
Canada	-0.29(0.013)***	-0.701(0.029)***	-0.047(0.037)	-0.116(0.091)
Canada (Ontario)	-0.295(0.016)***	-0.675(0.037)***	-0.156(0.037)***	-0.381(0.091)***
Canada (Quebec)	-0.274(0.018)***	-0.711(0.044)***	-0.079(0.073)	-0.21(0.191)
Chile	-0.169(0.015)***	-0.424(0.039)***	-0.125(0.05)*	-0.34(0.126)**
Egypt	-0.147(0.012)***	-0.345(0.029)***	-0.142(0.041)**	-0.342(0.098)***
England	-0.094(0.014)***	-0.329(0.053)***	-0.332(0.057)***	-1.262(0.133)***
Georgia	-0.219(0.019)***	-0.491(0.044)***	-0.153(0.042)***	-0.394(0.102)***
Hong Kong	-0.19(0.013)***	-0.562(0.038)***	-0.075(0.058)	-0.276(0.212)
Hungary	-0.181(0.014)***	-0.495(0.039)***	-0.143(0.044)**	-0.457(0.134)**
Iran, Islamic Republic of	-0.187(0.012)***	-0.49(0.031)***	-0.142(0.042)**	-0.403(0.114)***
Ireland	-0.178(0.013)***	-0.505(0.036)***	-0.044(0.028)	-0.172(0.107)
Israel	-0.088(0.01)***	-0.298(0.032)***	-0.089(0.024)***	-0.397(0.106)***
Italy	-0.273(0.019)***	-0.624(0.038)***	-0.022(0.033)	-0.052(0.078)
Japan	-0.17(0.012)***	-0.384(0.028)***	0.015(0.04)	0.04(0.107)
Jordan	-0.164(0.014)***	-0.375(0.032)***	-0.044(0.044)	-0.105(0.105)
Kazakhstan	-0.109(0.014)***	-0.333(0.041)***	-0.047(0.052)	-0.149(0.164)
Korea, Republic of	-0.062(0.016)***	-0.144(0.037)***	0.062(0.045)	0.174(0.123)
Kuwait	-0.102(0.02)***	-0.264(0.051)***	-0.012(0.07)	-0.033(0.186)
Lebanon	-0.115(0.02)***	-0.288(0.05)***	-0.067(0.053)	-0.184(0.135)
Lithuania	-0.25(0.017)***	-0.57(0.041)***	-0.12(0.044)**	-0.321(0.119)**
Malaysia	-0.142(0.01)***	-0.401(0.031)***	-0.388(0.043)***	-1.139(0.089)***
Malta	-0.153(0.012)***	-0.515(0.039)***	0.012(0.067)	0.041(0.236)
Morocco	-0.163(0.012)***	-0.367(0.026)***	-0.067(0.033)*	-0.156(0.078)*
New Zealand	-0.155(0.013)***	-0.458(0.04)***	-0.136(0.027)***	-0.599(0.11)***
Norway	-0.322(0.016)***	-0.678(0.033)***	-0.056(0.024)*	-0.14(0.065)*
Norway (8)	-0.322(0.017)***	-0.683(0.037)***	-0.083(0.029)**	-0.214(0.077)**

Oman	-0.173(0.014)***	-0.388(0.031)***	-0.043(0.03)	-0.112(0.075)
Qatar	-0.163(0.014)***	-0.432(0.039)***	-0.219(0.051)***	-0.606(0.136)***
Russian Federation	-0.234(0.013)***	-0.582(0.032)***	-0.088(0.055)	-0.219(0.134)
Saudi Arabia	-0.11(0.02)***	-0.258(0.045)***	-0.089(0.05)	-0.21(0.119)
Singapore	-0.127(0.008)***	-0.4(0.031)***	-0.43(0.041)***	-1.35(0.076)***
Slovenia	-0.234(0.019)***	-0.522(0.039)***	-0.04(0.025)	-0.103(0.066)
South Africa	-0.102(0.01)***	-0.309(0.031)***	-0.087(0.07)	-0.333(0.261)
Sweden	-0.273(0.016)***	-0.625(0.036)***	-0.128(0.052)*	-0.33(0.139)*
Taiwan	-0.199(0.016)***	-0.448(0.036)***	-0.015(0.039)	-0.049(0.124)
Thailand	-0.113(0.012)***	-0.317(0.036)***	-0.209(0.068)**	-0.651(0.174)***
Turkey	-0.262(0.013)***	-0.622(0.031)***	-0.241(0.046)***	-0.583(0.113)***
United Arab Emirates	-0.179(0.009)***	-0.518(0.025)***	-0.048(0.033)	-0.151(0.104)
United Arab Emirates (Abu Dhabi)	-0.167(0.012)***	-0.467(0.032)***	-0.114(0.063)	-0.375(0.199)
United Arab Emirates (Dubai)	-0.212(0.02)***	-0.592(0.055)***	-0.052(0.044)	-0.161(0.137)
United States	-0.154(0.007)***	-0.445(0.023)***	-0.221(0.028)***	-0.752(0.083)***

Note. $^{***}p < 0.001$, $^{**}p < 0.01$, $^{*}p < 0.05$.

Additional Analyses

Is there a Contextual Effect of Math Anxiety at the Country Level?

Description

Just as students are nested in education environment, education environments are nested into different countries. This additional level of nesting raises the possibility that between-country differences in math anxiety may predict student achievement. To investigate whether country-average math anxiety may affect student achievement, we estimated a simple three-level model for each database. This model will yield estimates of the individual effect, the education environment contextual effect and the country contextual effect. Specifically, we have the following model:

$$\text{L1: } Y_{i,j,k} = \pi_{0,j,k} + \pi_{1,j,k}\left(X_{i,j,k} - \bar{X}_{\cdot,\cdot,\cdot}\right) + e_{i,j,k}$$
$$\text{L2: } \pi_{0,j,k} = \beta_{0,0,k} + \beta_{0,1,k}\left(X_{\cdot,j,k} - \bar{X}_{\cdot,\cdot,\cdot}\right) + r_{0,j,k}$$
$$\text{L3: } \beta_{0,0,k} = \gamma_{0,0,0} + \gamma_{0,0,1}\left(X_{\cdot,\cdot,k} - \bar{X}_{\cdot,\cdot,\cdot}\right) + \mu_{0,0,k}$$

Math anxiety is grand-mean centered, and a manifest aggregation approach is utilized. As such, the higher-level regression coefficients are a direct estimation of the contextual effect that controls for lower-level variations (52, 53).

Results

Having found statistically significant between-country differences, we next ascertain whether country-average math anxiety may affect math achievement. We estimated a three-level model for each database. In the TIMSS Grade 4 sample, L1 math anxiety was statistically significant and negatively related to math achievement ($\pi_{1,j,k} = -.172, SE = .005, \Delta = .508$), the contextual effect of L2 math anxiety on math achievement was also statistically significant and negative ($\beta_{0,0,k} = -.136, SE = .019, \Delta = .441$), however, the contextual effect of L3 math anxiety on math achievement was not statistically significant ($\pi_{1,j,k} = -.020, SE = .076, \Delta = .064$).

In the TIMSS Grade 8 sample, L1 math anxiety was statistically significant and negatively related to math achievement ($\pi_{1,j,k} = -.139, SE = .007, \Delta = .450$), the contextual effect of L2 math anxiety on math achievement was also statistically significant and negative ($\beta_{0,0,k} =$

$-.090, SE = .013, \Delta = .349$), however, the contextual effect of L3 math anxiety on math achievement was not statistically significant ($\pi_{1,j,k} = -.009, SE = .078, \Delta = .030$).

In the PISA sample, L1 math anxiety was statistically significant and negatively related to math achievement ($\pi_{1,j,k} = -.146, SE = .006, \Delta = .404$), the contextual effect of L2 math anxiety on math achievement was also statistically significant and negative ($\beta_{0,0,k} = -.098, SE = .014, \Delta = .355$). Finally, the contextual effect of L3 math anxiety on math achievement was statistically significant ($\pi_{1,j,k} = -.222, SE = .043, \Delta = .624$).

In sum, results for L1 and L2 are consistent with the previous analyses – specifically, that there is a negative association between individual math anxiety and math achievement and a negative association between education environment-average math anxiety and math achievement. Consistent with previous analysis, the effects had a small to medium effect size (.349 – .508). Interestingly, the L3 contextual effect that associates between country-average math anxiety and math achievement was statistically significant for the PISA database but were not statistically significant for the TIMSS databases. In other words, results from the PISA database supported the notion that a country's average level of math anxiety is related to children's math achievement but results from the TIMSS databases did not support this hypothesis.

Are there Between-Country Variations in the Magnitude of the Individual and Contextual Effects?

Description

Another possible area of between-country differences in the relations between math anxiety and math achievement is between-country differences in the magnitude of the L1 individual effect and L2 contextual effect. To examine this, we modified the simple three-level model above to allow for the L1 and L2 effects to vary at the higher levels:

$$\text{L1: } Y_{i,j,k} = \pi_{0,j,k} + \pi_{1,j,k}\left(X_{i,j,k} - \bar{X}_{\cdot,\cdot,\cdot}\right) + e_{i,j,k}$$

$$\text{L2: } \pi_{0,j,k} = \beta_{0,0,k} + \beta_{0,1,k}\left(X_{\cdot,j,k} - \bar{X}_{\cdot,\cdot,\cdot}\right) + r_{0,j,k}$$

$$\pi_{1,j,k} = \beta_{1,0,k} + \mathrm{r}_{1,j,k}$$

$$\text{L3: } \beta_{0,0,k} = \gamma_{0,0,0} + \gamma_{0,0,1}\left(X_{\cdot,\cdot,k} - \bar{X}_{\cdot,\cdot,\cdot}\right) + \mu_{0,0,k}$$

$$\beta_{0,1,k} = \gamma_{0,1,0} + u_{0,1,k}$$

$$\beta_{1,0,k} = \gamma_{1,0,0} + u_{1,0,k}$$

A significant L1 random slope at L2 ($r_{1,j,k}$) or L3 ($u_{1,0,k}$) would suggest that the magnitude of relation between individual math anxiety and math achievement differ as a function of membership in the education environment or country, or both. Relatedly, a significant L2 random slope at L3 ($u_{0,1,k}$) would suggest the relation between environment-average math anxiety and math achievement differ as a function of membership in country.

Results

We have shown in the previous analyses that there are between-country differences in the magnitude of the L1 individual effects and L2 contextual effects. We next quantify these between-country differences by estimating a three-level model with random-slopes. Results are presented in **Table S5**.

Results indicate that magnitude of effect of individual math anxiety on math achievement ($\gamma_{1,0,0}$) to significantly differ at L2 ($r_{1,j,k}$) and L3 ($u_{1,0,k}$) for all three databases. This suggest that the effect that individual math anxiety has on math achievement differ depending on the education environment and country membership. There was a significant average L1 individual effect of math anxiety on math achievement for the TIMSS Grade 4 ($\gamma_{1,0,0} = -.179, SE = .005, \Delta = .531$), TIMSS Grade 8 ($\gamma_{1,0,0} = -.143, SE = .007, \Delta = .462$), and PISA ($\gamma_{1,0,0} = -.139, SE = .007, \Delta = .383$). To ascertain the typical range of values that the individual effects may differ between countries and education environments, we calculated a 95% plausible value range (54), which theoretically encompasses the magnitude of the individual effect for 95% of education environments and countries (55). The 95% plausible value range was -.286 to -.072, -.267 to -.019 and -.291 to .013 for TIMSS Grade 4, TIMSS Grade 8 and PISA, respectively.

Similarly, we find that the magnitude of education environment-average math anxiety on math achievement ($\gamma_{0,1,0}$) to significantly differ at L3 ($u_{0,1,k}$) for all three databases. This suggest that the effect that environment-average math anxiety has on math achievement differ depending on country membership. There was a significant average L2 contextual effect for the TIMSS Grade 4 ($\gamma_{0,1,0} = -.117, SE = .014, \Delta = .382$), TIMSS Grade 8 ($\gamma_{0,1,0} = -.102, SE = .015, \Delta = .395$), and PISA ($\gamma_{0,1,0} = -.129, SE = .013, \Delta = .466$). The 95% plausible value range, the

degree of variability between countries for the magnitude of the contextual effect was -.315 to .081, -.290 to .085 and -.317 to .059 for TIMSS Grade 4, TIMSS Grade 8 and PISA, respectively.

Finally, we find that the effect that country-average math anxiety has on math achievement ($\gamma_{0,0,1}$) was insignificant for the TIMSS Grade 4 ($\gamma_{0,0,1} = -.018, SE = .077, \Delta = .058$), and Grade 8 ($\gamma_{0,0,1} = -.009, SE = .076, \Delta = .021$), but was statistically significant for the PISA ($\gamma_{0,0,1} = -.215, SE = .042, \Delta = .604$).

In sum, results from the three-level model with random slopes revealed that on average, there is a L1 individual effect of math anxiety on math achievement. Extending the previous results, we find that both the education environment membership and country membership contributes to variability in how individual's math anxiety may affect math achievement. Similarly, we find that on average there is a L2 contextual effect of math anxiety on math achievement. Further, we find that contextual effect depends on country membership.

Table S5

Results for the Three-Level Model with Random-Slopes

	TIMSS Grade 4	*TIMSS Grade 8*	*PISA*
	Coefficient (SE)	*Coefficient (SE)*	*Coefficient (SE)*
Fixed Effect			
Average Initial Math Achievement, $\gamma_{0,0,0}$	.047(.084)	.022(.088)	-.101(.054)
Individual Math Anxiety, $\gamma_{1,0,0}$	-.179(.005)***	-.143(.007)***	-.139(.007)***
Education Environment Math Anxiety, $\gamma_{0,1,0}$	-.117(.014)***	-.102(.015)***	-.129(.013)***
Country Math Anxiety, $\gamma_{0,0,1}$	-.018(.077)	-.009(.076)	-.215(.042)***
Random Effect			
Math Achievement L1 Residual, $e_{i,j,k}$	.406(.015)***	.348(.020)***	.430(.017)***
Math Achievement L2 Residual, $r_{0,j,k}$	.189(.025)***	.264(.022)***	.261(.019)***
Math Achievement L3 Residual, $\mu_{0,0,k}$	.322(.070)***	.330(.071)***	.175(.037)***
Individual Math Anxiety at L2, $r_{1,j,k}$	.002(.001)**	.002(.000)***	.003(.001)***
Individual Math Anxiety at L3, $u_{1,0,k}$	.001(.000)***	.002(.000)***	.003(.000)***

Education Environment Math Anxiety at L3, $u_{0,1,k}$	.010(.003)***	.009(.003)**	.009(.002)***
Average Effect Size (Δ)			
Individual Math Anxiety	.531	.462	.383
Education Environment Math Anxiety	.382	.395	.466
Country Math Anxiety	.058	.021	.604

Note. $^{***}p < 0.001$, $^{**}p < 0.01$, $^{*}p < 0.05$.

SI References

1. OECD, *PISA 2012 technical report* (OECD publishing Paris, 2014).

2. W. Van den Noortgate, M.-C. Opdenakker, P. Onghena, The effects of ignoring a level in multilevel analysis. *School Effectiveness and School Improvement* **16**, 281–303 (2005).

3. M. O. Martin, I. V. Mullis, M. Hooper, Methods and procedures in TIMSS 2015. *TIMSS & PIRLS International Study Center, Lynch School of Education, Boston College and International Association for the Evaluation of Educational Achievement (IEA)* (2016).

4. A. C. Carle, Fitting multilevel models in complex survey data with design weights: Recommendations. *BMC medical research methodology* **9**, 49 (2009).

5. L. Rutkowski, E. Gonzalez, M. Joncas, M. von Davier, International large-scale assessment data: Issues in secondary analysis and reporting. *Educational Researcher* **39**, 142–151 (2010).

6. B. Nagengast, H. W. Marsh, The negative effect of school-average ability on science self-concept in the UK, the UK countries and the world: the Big-Fish-Little-Pond-Effect for PISA 2006. *Educational Psychology* **31**, 629–656 (2011).

7. L. M. Stapleton, An assessment of practical solutions for structural equation modeling with complex sample data. *Structural Equation Modeling* **13**, 28–58 (2006).

8. United Nations Development Program, Human Development Data (1990-2018). *Human Development Data (1990-2018)* (2015) (October 5, 2020).

9. G. H. Hofstede, G. J. Hofstede, M. Minkov, *Cultures and organizations: Software of the mind*, 3rd Ed. (Mcgraw-hill New York, 2010).

10. K. H. Dew, J. P. Galassi, Mathematics anxiety: Some basic issues. *Journal of Counseling Psychology* **30**, 443 (1983).

11. A. M. Ferguson, E. A. Maloney, J. Fugelsang, E. F. Risko, On the relation between math and spatial ability: The case of math anxiety. *Learning and Individual Differences* **39**, 1–12 (2015).

12. R. Hembree, The nature, effects, and relief of mathematics anxiety. *Journal for research in mathematics education*, 33–46 (1990).

13. H. Miller, J. Bichsel, Anxiety, working memory, gender, and math performance. *Personality and Individual Differences* **37**, 591–606 (2004).

14. T. Woodard, The Effects of Math Anxiety on Post-Secondary Developmental Students as Related to Achievement, Gender, and Age. *Inquiry* **9**, n1 (2004).

15. M. J. Gierl, J. Bisanz, Anxieties and attitudes related to mathematics in grades 3 and 6. *The Journal of experimental education* **63**, 139–158 (1995).

16. R. R. Harari, R. K. Vukovic, S. P. Bailey, Mathematics anxiety in young children: An exploratory study. *The Journal of experimental education* **81**, 538–555 (2013).

17. G. Ramirez, E. A. Gunderson, S. C. Levine, S. L. Beilock, Math anxiety, working memory, and math achievement in early elementary school. *Journal of Cognition and Development* **14**, 187–202 (2013).

18. M. S. Griggs, S. E. Rimm-Kaufman, E. G. Merritt, C. L. Patton, The Responsive Classroom approach and fifth grade students' math and science anxiety and self-efficacy. *School Psychology Quarterly* **28**, 360 (2013).

19. E. Satake, P. P. Amato, Mathematics anxiety and achievement among Japanese elementary school students. *Educational and Psychological Measurement* **55**, 1000–1007 (1995).

20. F. Yüksel-Şahin, Mathematics anxiety among 4th and 5th grade Turkish elementary school students. *International Electronic Journal of Mathematics Education* **3**, 179–192 (2008).

21. S. L. Beilock, E. A. Gunderson, G. Ramirez, S. C. Levine, Female teachers' math anxiety affects girls' math achievement. *Proceedings of the National Academy of Sciences* **107**, 1860–1863 (2010).

22. M. W. Schaeffer, *et al.*, Elementary school teachers' math anxiety and students' math learning: A large-scale replication. *Developmental Science,* e13080 (2020).

23. W. Ahmed, A. Minnaert, H. Kuyper, G. van der Werf, Reciprocal relationships between math self-concept and math anxiety. *Learning and individual differences* **22**, 385–389 (2012).

24. J. Lee, Universals and specifics of math self-concept, math self-efficacy, and math anxiety across 41 PISA 2003 participating countries. *Learning and individual differences* **19**, 355–365 (2009).

25. L. A. Fast, *et al.*, Does math self-efficacy mediate the effect of the perceived classroom environment on standardized math test performance? *Journal of Educational Psychology* **102**, 729 (2010).

26. J. Radišić, M. Videnović, A. Baucal, Math anxiety—contributing school and individual level factors. *European Journal of Psychology of Education* **30**, 1–20 (2015).

27. D. Aslan, A COMPARISON OF PRE-AND IN-SERVICE PRESCHOOL TEACHERS'MATHEMATICAL ANXIETY AND BELIEFS ABOUT MATHEMATICS FOR YOUNG CHILDREN. *Academic Research International* **4**, 225 (2013).

28. Y. Lu, Q. Li, H. Patrick, P. Mantzicopoulos, "Math Gives Me a Tummy Ache!" Mathematics Anxiety in Kindergarten. *The Journal of Experimental Education*, 1–17 (2019).

29. D. J. Stipek, R. H. Ryan, Economically disadvantaged preschoolers: Ready to learn but further to go. *Developmental psychology* **33**, 711 (1997).

30. T. Berkowitz, *et al.*, Math at home adds up to achievement in school. *Science* **350**, 196–198 (2015).

31. M. M. Jameson, Contextual factors related to math anxiety in second-grade children. *The Journal of Experimental Education* **82**, 518–536 (2014).

32. J. R. Cheema, K. Sheridan, Time spent on homework, mathematics anxiety and mathematics achievement: Evidence from a US sample. *Issues in Educational Research* **25**, 246 (2015).

33. E. A. Maloney, G. Ramirez, E. A. Gunderson, S. C. Levine, S. L. Beilock, Intergenerational effects of parents' math anxiety on children's math achievement and anxiety. *Psychological Science* **26**, 1480–1488 (2015).

34. K. Supekar, T. Iuculano, L. Chen, V. Menon, Remediation of childhood math anxiety and associated neural circuits through cognitive tutoring. *Journal of Neuroscience* **35**, 12574–12583 (2015).

35. B. Onslow, Improving the attitude of students and parents through family involvement in mathematics. *Mathematics Education Research Journal* **4**, 24–31 (1993).

36. J. E. Parsons, T. F. Adler, C. M. Kaczala, Socialization of achievement attitudes and beliefs: Parental influences. *Child development*, 310–321 (1982).

37. A. Soni, S. Kumari, The role of parental math anxiety and math attitude in their children's math achievement. *International Journal of Science and Mathematics Education* **15**, 331–347 (2017).

38. N. F. Baya'a, Mathematics anxiety, mathematics achievement, gender, and socio-economic status among Arab secondary students in Israel. *International Journal of Mathematical Education in Science and Technology* **21**, 319–324 (1990).

39. J. R. Cheema, G. Galluzzo, Analyzing the gender gap in math achievement: Evidence from a large-scale US sample. *Research in Education* **90**, 98–112 (2013).

40. S. K. Geyik, The effects of parents' socio economic status on mathematics anxiety among social sciences students in turkey. *International Journal of Education and Research* **3**, 311–324 (2015).

41. W. Ahmed, Developmental trajectories of math anxiety during adolescence: Associations with STEM career choice. *Journal of adolescence* **67**, 158–166 (2018).

42. S. R. Sirin, Socioeconomic status and academic achievement: A meta-analytic review of research. *Review of educational research* **75**, 417–453 (2005).

43. J. Boaler, D. Wiliam, M. Brown, Students' experiences of ability grouping-disaffection, polarisation and the construction of failure. *British educational research journal* **26**, 631–648 (2000).

44. S. Catsambis, L. Mulkey, R. Crain, For better or for worse? A nationwide study of the social psychological effects of gender and ability grouping in mathematics. *Social Psychology of Education* **5**, 83–115 (2001).

45. E. Novak, J. L. Tassell, Studying preservice teacher math anxiety and mathematics performance in geometry, word, and non-word problem solving. *Learning and Individual Differences* **54**, 20–29 (2017).

46. G. Ramirez, S. Y. Hooper, N. B. Kersting, R. Ferguson, D. Yeager, Teacher math anxiety relates to adolescent students' math achievement. *AERA open* **4**, 2332858418756052 (2018).

47. M. Bursal, L. Paznokas, Mathematics anxiety and preservice elementary teachers' confidence to teach mathematics and science. *School Science and Mathematics* **106**, 173–180 (2006).

48. E. Geist, Math anxiety and the "math gap": How attitudes toward mathematics disadvantages students as early as preschool. *Education* **135**, 328–336 (2015).

49. C. Cornell, I hate math! I couldn't learn it, and I can't teach it! *Childhood education* **75**, 225 (1999).

50. C. D. Jackson, R. J. Leffingwell, The role of instructors in creating math anxiety in students from kindergarten through college. *The Mathematics Teacher* **92**, 583–586 (1999).

51. A. E. Foley, *et al.*, The math anxiety-performance link: A global phenomenon. *Current Directions in Psychological Science* **26**, 52–58 (2017).

52. A. M. Brincks, *et al.*, Centering predictor variables in three-level contextual models. *Multivariate behavioral research* **52**, 149–163 (2017).

53. C. K. Enders, D. Tofighi, Centering predictor variables in cross-sectional multilevel models: a new look at an old issue. *Psychological methods* **12**, 121 (2007).

54. S. W. Raudenbush, A. S. Bryk, *Hierarchical linear models: Applications and data analysis methods* (Sage, 2002).

55. J. Lorah, Effect size measures for multilevel models: Definition, interpretation, and TIMSS example. *Large-Scale Assessments in Education* **6**, 8 (2018).
